# The Tiara Nickel Cluster Story from Theory to Catalytic Applications

**DOI:** 10.1021/prechem.4c00087

**Published:** 2025-01-09

**Authors:** Piracha Sanwal, Xinrui Gu, Yifei Zhang, Gao Li

**Affiliations:** † Institute of Catalysis for Energy and Environment, College of Chemistry and Chemical Engineering, Shenyang Normal University, Shenyang 110034, China; ‡ State Key Laboratory of Catalysis, Dalian Institute of Chemical Physics, Chinese Academy of Sciences, Dalian 116023, China; § University of Chinese Academy of Sciences, Beijing 100049, China

**Keywords:** Tiara nickel cluster, photocatalysis, hydrogen evolution reaction, oxygen evolution reaction, density functional theory

## Abstract

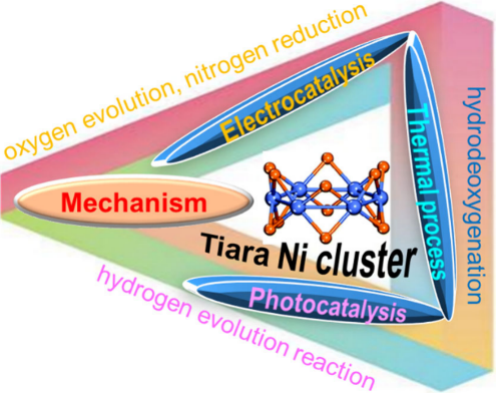

As a transition material between bulk materials and individual atoms, nickel clusters have interesting electrical and structural characteristics that could make them useful as catalysts. To examine the catalytic efficiency of nickel clusters in different applications, this Review combines experimental techniques with density functional theory (DFT). Researchers have shown that nickel clusters can activate and alter tiny molecules like CO, NO, and H_2_ through DFT studies that delve deeply into their electronic structures, adsorption mechanisms, and stability. These findings lay the groundwork for the development of effective catalysts for various processes. Nickel clusters considerably improve the hydrogen evolution reaction (HER), indicating their promise for renewable energy conversion. Furthermore, electrocatalysis for the oxygen evolution reaction (OER) showcases the electrochemical performance of nickel clusters, demonstrating their stability and efficiency. The adaptability of nickel clusters is further demonstrated by their use in nitrogen reduction to ammonia. Experimental data confirm that these clusters are good catalysts for this crucial industrial activity. Hydrocarbon reforming and pollutant degradation are two areas in which research has shown that nickel clusters can be useful in thermal reactions. X-ray absorption spectroscopy (XAS) and environmental transmission electron microscopy (ETEM) are examples of in situ characterization techniques that support theoretical predictions by providing real-time insights into the structural alterations and active sites of nickel clusters during these processes. Preparing the way for future research and practical applications in energy and environmental technologies, this thorough study highlights the great potential of nickel clusters in constructing sustainable and efficient catalytic systems.

## 1 Introduction

Out of all transition metal clusters that could serve as prototypes for the study of molecular adsorption, particular attention is paid to the ones that are generated by nickel, Ni. This is because nickel has high melting temperature (appropriate for typical application circumstances), is reasonably priced, has strong activity even with low reactive substrates, and can reach unusual oxidations.^1−4^ In cluster form, Ni demonstrates a high surface/volume ratio, active adsorption, reaction sites, a wide spectrum of possible isomers, and ease of structural rearrangements. Because their sizes, shapes, and interacting systems may all be used to modify their physical and chemical characteristics, tiny Ni clusters have generated a lot of attention.^5,6^ As prototype systems to study the molecular adsorption process, Ni clusters are thus very important to a deeper understanding of the interaction between Ni clusters and diatomic molecules.^7,8^ The unique structural and physicochemical properties of nickel clusters, particularly at the nanoscale, make them highly important in catalytic processes.^9^ Depending on the number of atoms and the synthesis method, these clusters can exist at the atomic level in a range of geometric shapes, such as tetrahedral, octahedral, and more complex structures.^10^ Because of their small size and high surface-to-volume ratio, Ni clusters have a high density of reactive sites, which significantly boosts their catalytic activity. The electronic structure of these clusters is very different from that of bulk nickel due to quantum size effects, which lead to quantization of the energy levels and a rise in reactivity.^11^ Ni clusters structurally rearrange in response to outside stimuli like pressure or temperature, which has an additional impact on their catalytic performance. The physicochemical properties of Ni clusters, such as their electronic structure, charge distribution, and reactivity, make them ideal for a range of catalytic applications.^12^ The electrical structure and adsorption patterns of small nickel clusters (Ni_2_–Ni_19_) have been clarified by DFT and other computational methods, which have also shown that these clusters have the ability to activate small molecules like hydrogen, NO, and CO.^13,14^

The study of nickel cluster characteristics has greatly benefited by the use of density functional theory (DFT).^15−19^ Deep insights into the electronic structure, stability, and catalytic processes of these clusters may be gained using DFT calculations.^20−23^ The potential of different compounds in catalytic processes has been shown by theoretical research that has clarified their adsorption behaviors on nickel clusters.^24,25^ DFT investigations, for example, on tiny nickel clusters, Ni_2_–Ni_19_, have yielded important insights into their electrical characteristics and structural dynamics.^14,26−28^ DFT has also been used to investigate the potential for catalysis and interaction processes of tiny molecules such as CO, NO, and SO when they adsorb on nickel clusters.^29−31^ These investigations have shown that nickel clusters are very useful for catalytic applications because of their ability to efficiently activate and change tiny molecules.^32−39^ Furthermore, DFT has been widely used to investigate the adsorption and activation of hydrogen (H_2_) on both free and graphene-supported nickel clusters. These theoretical investigations have shown the usefulness of nickel clusters in sustainable energy technologies by demonstrating their ability to function as effective catalysts for hydrogen evolution events.^40−46^

By offering useful insights into the synthesis, characterization, and catalytic performance of nickel clusters, experimental research enhances theoretical investigations.^47−49^ A range of experimental methodologies have been used to synthesize nickel clusters with atomic precision and assess their catalytic characteristics.^9,50,51^ One noteworthy technique is the cosynthesis of nickel clusters with ligands containing sulfur, which results in the production of distinct clusters like Ni_4_(SR)_8_.^52−54^ Using this method, we investigated the optical and catalytic characteristics of these clusters, showing promise for a range of catalytic processes. Additionally, nickel cluster catalytic applications in hydrogen evolution reactions and electrochemical processes have been the subject of experimental research.^10,55^ Ni_6_(SC_2_H_4_Ph)_12_ clusters, for example, supported on g-C_3_N_4_ nanosheets have shown improved H_2_ evolution by photocatalysis, underscoring its usefulness in sustainable energy applications.^56−59^ Other experimental studies have used nickel clusters to study the electrocatalytic oxygen evolution and hydrogenation reactions, demonstrating the stability and efficiency of these processes under various circumstances.^60−62^

To further enhance their catalytic efficiency, the synthesis of nickel clusters for the reduction of nitrogen to ammonia has been investigated.^63,64^ These experiments have shown that nickel clusters are useful for catalytic activities such as hydrogen production and electrochemical sensing.^65^ A comprehensive knowledge of the characteristics and uses of nickel clusters is made possible by the combination of DFT and experimental research. DFT provides a comprehensive understanding of the electrical and structural properties of these clusters, and experimental studies verify and investigate useful applications. When combined, these methods open the door to the creation of effective nickel-based catalysts for a range of industrial and environmental uses.

## 2 Mechanism

### 2.1 DFT-PBE+D3 Calculations

**Figure 1:**
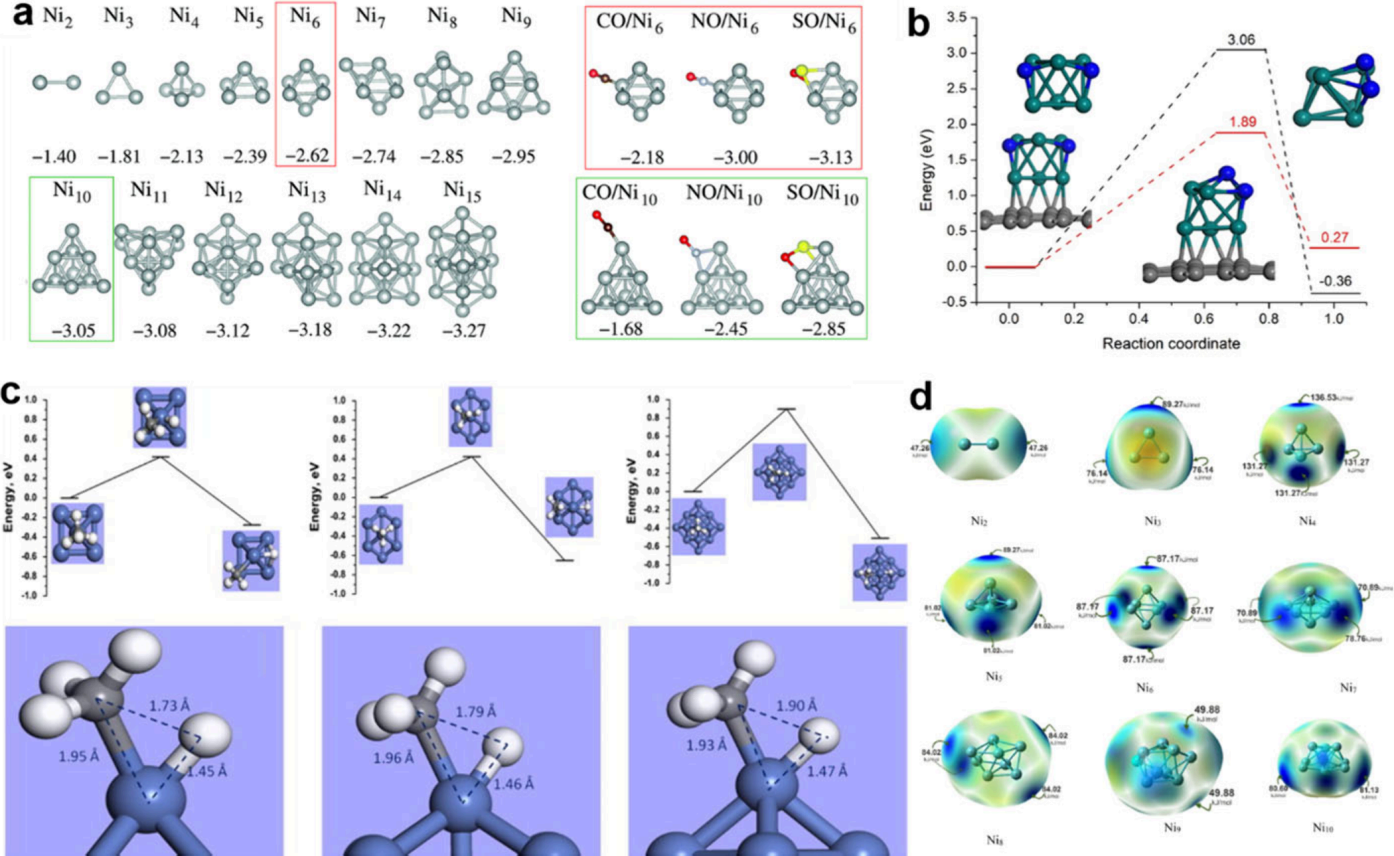
(a) The lowest energy configurations of Ni_*n*_ (*n* = 2–15) clusters. Reproduced with permission from ref (75). Copyright 2020, Royal Society of Chemistry. (b) Calculated reaction paths and corresponding geometries of initial and TSs of nonadjacent. Reproduced with permission from ref (38). Copyright 2021, Elsevier B.V. (c) ESP spanning clusters with various arrangements of TSs: Ni_6_, Ni_13_ and Ni_19_. Reproduced with permission from ref (76). Copyright 2015, Elsevier B.V. (d) ESP over the Ni_*n*_ (*n* = 2–10) clusters. Reproduced with permission from ref (77). Copyright 2018, Elsevier B.V.

As well as their structural and electrical characteristics, the lowest energy structures of Ni_*n*_ (*n* = 2–15) and their binding energies per atom, Eb, Figure 1a, are employed by a different method (DFT-PBE+D3) and a computational package (VASP) by Song et al.^57^ and Chaves et al.^66,67^ It revealed excellent agreement between our lowest energy designs and their features. Square or triangular bipyramids with tetrahedral and octahedral motifs serve as the foundation for the development pattern.^68^ Ni atom growth increases Eb (Figure 1a), becoming closer to bulk cohesive energy (4.44 eV/atom in experiments vs −4.76 eV/atom in calculations).^69^ Since the d-band average values essentially grow linearly with atom number, lower-coordinated atoms are characterized by constricted bonds, which increase binding energy per atom. When structural transitions take place between tetrahedral and octahedral patterns (and vice versa), as for *n* = 5–6 and *n* = 9–10, deviations from linear behavior occur. ECN values also rise with the number of atoms. With an increase in the number of Ni atoms (*n*), the magnetic transition (mT) values average 0.89 μB per Ni atom. Moreover, a plateau with the same mT value (8.0 μB) is shown for Ni_6_ through Ni_10_. Same as the bulk phase, our nickel clusters exhibit ferromagnetism.^70−73^

### 2.2 Decomposition of NH_3_ on Ni_6_ Clusters and Ni_6_@graphene

Furthermore, Figure 1b shows the reaction pathway of N + N recombination in the absence of proximity between the two N atoms. Similarly, Ni_6_@graphene is more prevalent than the Ni_6_ cluster, and the results are comparable. Just like previous theoretical studies on the Ni(111) surface, the rate-limiting mechanism is the N + N recombination, which has higher barriers than the dehydrogenation of NH_2_–H on Ni_6_ cluster and Ni_6_@graphene.^25^ A computed activation energy of 1.47 eV for the Ni_6_@graphene system is lower than 1.86 eV^25^ for the Ni(111) surface and somewhat higher than 1.32 eV^74^ for Ru_6_@CNT, suggesting a strong catalytic activity for ammonia breakdown.

### 2.3 Activation of Energy Barrier

Only the most stable product state configuration transition state (TS) is used to examine size’s influence on TSs. Figure 1c shows the TS geometries of the activation energy barriers. For clarification, the adsorbed condition and the energy barrier calculation reference is a bare Ni cluster distant from CH_4_.^78^ The C–H bond distance at the TS rises from 1.73 to 1.90 Å while transitioning from Ni_6_ to Ni_19_ clusters. Ni_13_ has higher reaction energies and exothermicity than Ni_6_, showing that even though they face the same obstacle, products are better stabilized with Ni_13_.^79,80^ The barrier for Ni_6_ and Ni_13_ NCs is 0.42 eV, but the barrier for Ni_19_ is 0.90 eV, more than double that of the other two. Ni_6_ and Ni_13_ have very low barrier energies compared to gas-phase reactants. Electrostatic potential (ESP) maps, which show molecular charge distributions, assist in identifying reactive sites.^81^ The ESP map of Ni_*n*_ (*n* = 2–10) cluster charge distributions was depicted in Figure 1d. Ni cluster surface ESP maximum values range from 47.26 (Ni_2_) to 136.53 kJ/mol (Ni_4_). The locations that exhibit the highest levels of electrostatic potential are regarded as active sites. Active locations have the highest ESP.^14^

**Figure 2:**
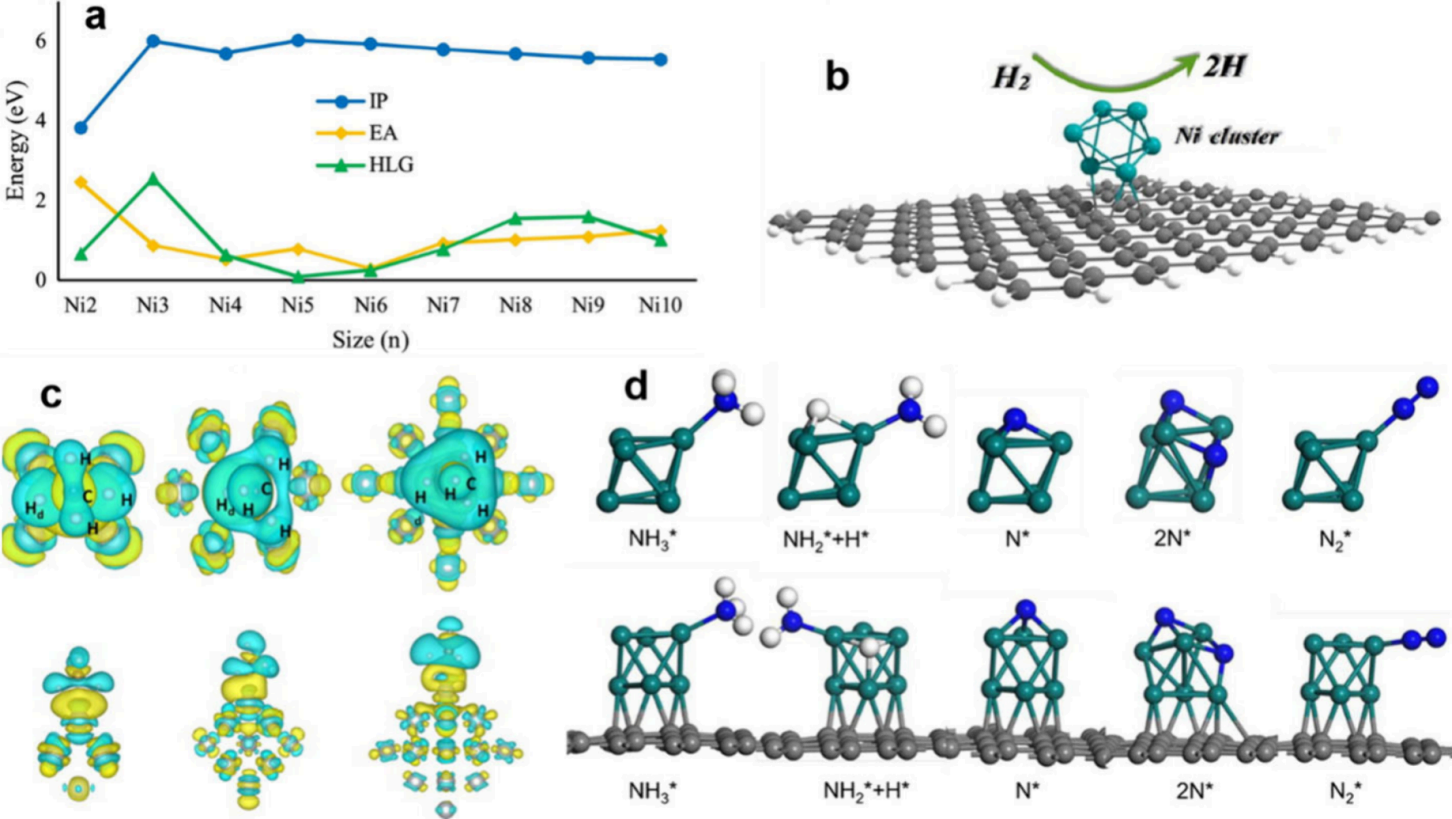
(a) HLG, IP and EA of Ni_*n*_ (*n* = 2–10) clusters. (b) Free and graphene-supported Ni cluster H_2_ adsorption. Reproduced with permission from ref (77). Copyright 2018, Elsevier B.V. (c) CCD plots of CH_4_ adsorbed on Ni_6_, Ni_13_ and Ni_19_. Reproduced with permission from ref (76). Copyright 2015, Elsevier B.V. (d) Ni_6_ cluster and Ni_6_@graphene species-related NH_3_ breakdown adsorption geometries. Reproduced with permission from ref (38). Copyright 2021, Elsevier B.V.

Plots of the Ni clusters’ IP, EA and HLG are displayed in Figure 2a. As a result, the Ni_2_ cluster has the lowest IP value, whereas the Ni_3_ cluster has the greatest IP value.^82^ It was discovered that when the number of Ni atoms in the Ni_*n*_ clusters increased, the IP dropped. As a result, ionizing an electron in the Ni_3_ cluster is more challenging than in other clusters. The Ni_2_ cluster is associated with the highest EA. As a result, compared to other clusters, this one can more easily absorb one electron to generate a radical anion.^83^ A DFT analysis of the stability and reactivity of graphene-supported Ni clusters concerning cluster size is shown in Figure 2b. The dissociative adsorption of the hydrogen molecule is the first stage of hydrogenation. As a result, we investigate the hydrogen atoms that have been adsorbed on these clusters. Additionally, the correctness and dependability of several DFT-D functionals for figuring out the geometry and electrical characteristics of clusters were assessed.^77^

### 2.4 Charge Density Difference

Charge density differential (CDD) plots were used to explore how CH_4_ adsorption influences the electron density. Figure 2c shows methane adsorption CDD maps of several clusters. Notable depletion was seen around the H atoms of the adsorbing CH_4_ molecule, and there was also evidence of charge buildup between the top nickel atom and the binding carbon atom. Charge transport from CH_4_ to the cluster is shown by the CDD graphs. It has already been noted that CH_4_ dissociates on Pt surfaces.^84^ Polarization away from the surface seems to reduce metal-molecule Pauli repulsion. As the cluster grows, the charge density distribution extends less to all its atoms, indicating that methane adsorption causes only localized charge shifts, Figure 2c. CDD graph of methane was adsorbed on Ni_6_, Ni_13_ and Ni_19_.^14^

## 3 Catalytic Applications

### 3.1 Photocatalysis

#### 3.1.1 Decipherment of Ni_6_ Cluster Structure

**Figure 3:**
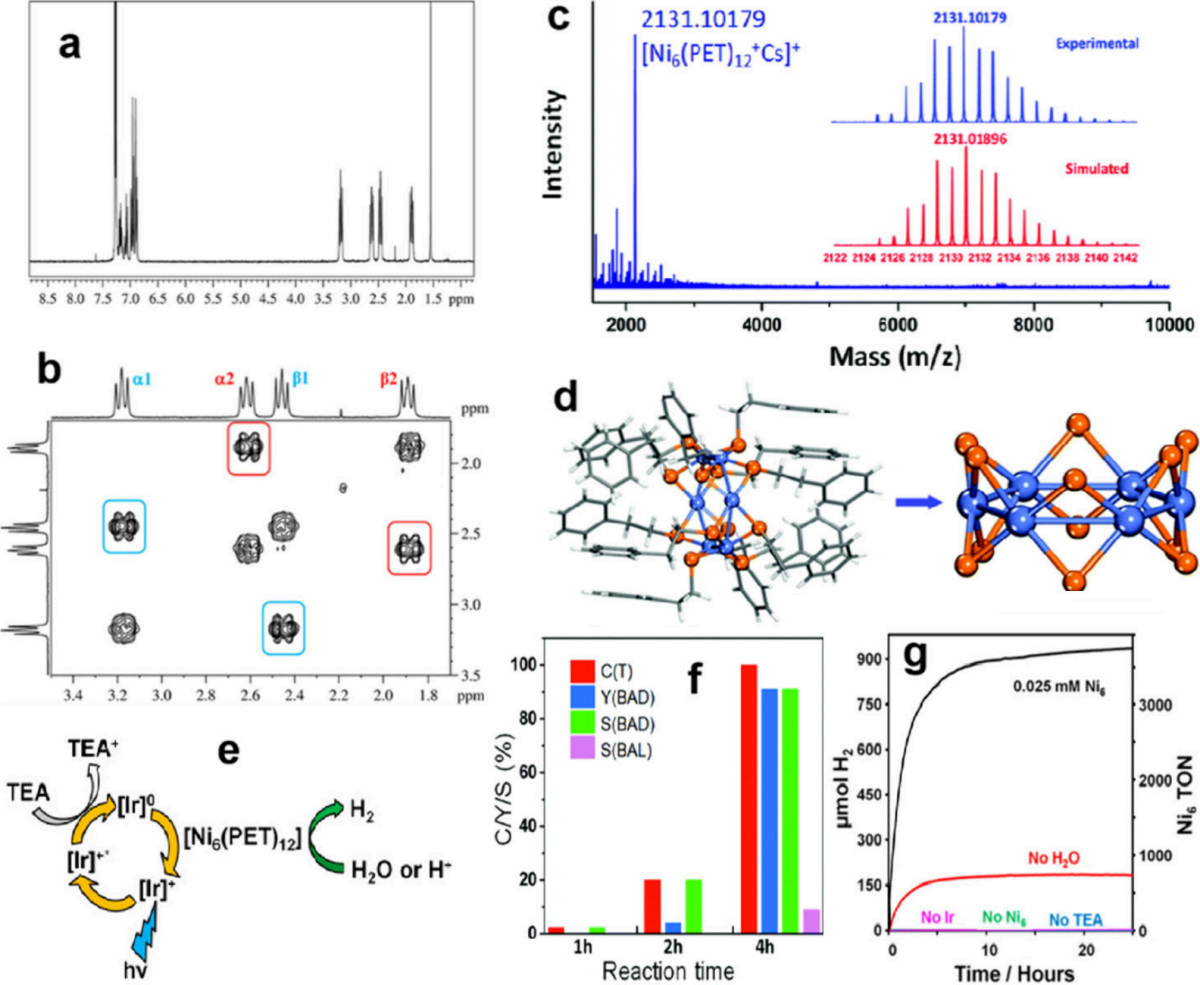
(a) ^1^H NMR and (b) 2D COSY spectra of Ni_6_(PET)_12_. (c) ESI-MS spectrum and (d) structure of Ni_6_(PET)_12_. (e) A nickel cluster-based photocatalytic hydrogen generation system. (f) Relationship between response time and the distribution of products. Reproduced with permission from ref (9). Copyright 2019, John Wiley & Sons. (g) Photocatalytic hydrogen production performance of Ni_6_(PET)_12_ as a WRC in comparison to controlled conditions. Reproduced with permission from ref (90). Copyright 2013, American Chemical Society.

The metallocrown-like structure was revealed by single crystal X-rays, which is consisted of six nickel atoms arranged in a hexagonal ring and six sulfur atoms positioned above and below the plane. As shown in Figure 3a, four aliphatic triplets derived from ^1^H NMR in CDCl_3_ are shown. The α-methylene (S-linked) protons are responsible for the peaks at 3.18 and 2.62 ppm, whereas the β-methylene protons are the ones responsible for the peaks at 2.46 and 1.89 ppm.^85^ These two peaks are formed when the ligands, because of their steric bulk, rotate axially and equatorially around the hexameric core.^86^ As depicted in Figure 3b, the results of a 2D COSY experiment confirmed the presence of two protons, classifying them as groups 1 and 2, which may indicate two distinct ligand environments.^9^ Gold clusters shielded by PET (phenylethanethiol) and [M(SR)_2_]_6_ complexes (where M = Ni, Pd, etc.) exhibit comparable structures. The as-prepared cluster’s positive-ion ESI-MS spectra reveal a notable peak at *m*/*z* 2131.10, which corresponds to the nickel hexametric adduct [Ni_6_(PET)_12_+Cs]^+^. The cluster solution’s neutral Ni_6_(PET)_12_ group was charged with cesium acetate before ESI-MS measurement, confirming the validity of the Ni_6_(PET)_12_ formula since all isotope peaks align with the simulations. The single-crystal structure of the Ni_6_(PET)_12_ cluster is shown in Figure 3d, which consists of 12 ligands around a hexagonal ring of six Ni atoms. Above and below the plane, the sulfur-bonded ligands stabilize six Ni atoms.^9^

However, the vial without water produced 700 turnovers. According to the established chemical route, consecutive electron transfers from the TEA liberate two protons. A few micrograms of trace water in the solvent may yield 150 μmol H_2_.^87^ The quick oxidation of TEA and the strong oxidizing excited state PS (E_*red_ = +0.77 V) cause reductive quenching, Figure 3e, which was supported by dynamic quenching research employing the Ir PS and Ni cluster. Ir^III^ PS/Ni catalyst pair exhibited a quenching constant of *k*_q_ = 3.7 × 10^9^ M^–1^ s^–1^, as determined from the Stern–Volmer plot. The Ni_6_ catalyst exhibits a smaller *k*_q_ compared to TEA, despite the TEA concentration being 1000 times higher in photoreactions, suggesting the occurrence of reductive quenching. The electrochemical HER results revealed that Ni^II^ undergoes a one-electron reduction, facilitated by electron transfer from the reduced photosensitizer, Ir^0^ and subsequent protonation. This process likely leads to the formation of a hydride intermediate. The input proton transforms, resulting in the production of H_2_.^88^

Figure 3f shows the reaction time-dependent product distribution of Ni_6_(PET)_12_ clusters in H_2_O_2_ at 80 °C. The other catalysts in earlier research showed strong benzaldehyde selectivity at high temperatures (160–400 °C). This catalyst demonstrated limited catalytic activity in the presence of oxygen under moderate conditions (80 °C, atmospheric pressure).^89^ The nickel cluster was used in a molecular system with [Ir(F-mppy)_2_(dtbbpy)](PF_6_) as the PS to achieve photocatalytic water reduction. Figure 3g shows the molecular structures of two photoreaction complexes using TEA as sacrificial reductant. In the first test, reaction vials contained 0.3 mM PS and 0.025 mM catalyst using PS, WRC, water, or TEA as controls. Depicted in Figure 3g are the systems’ H_2_ evolution traces as well as catalyst turnover numbers. Little hydrogen was generated when all of the components were presented in the control experiment.^90^

#### 3.1.2 Hydrogen Evolution Reaction (HER)

**Figure 4:**
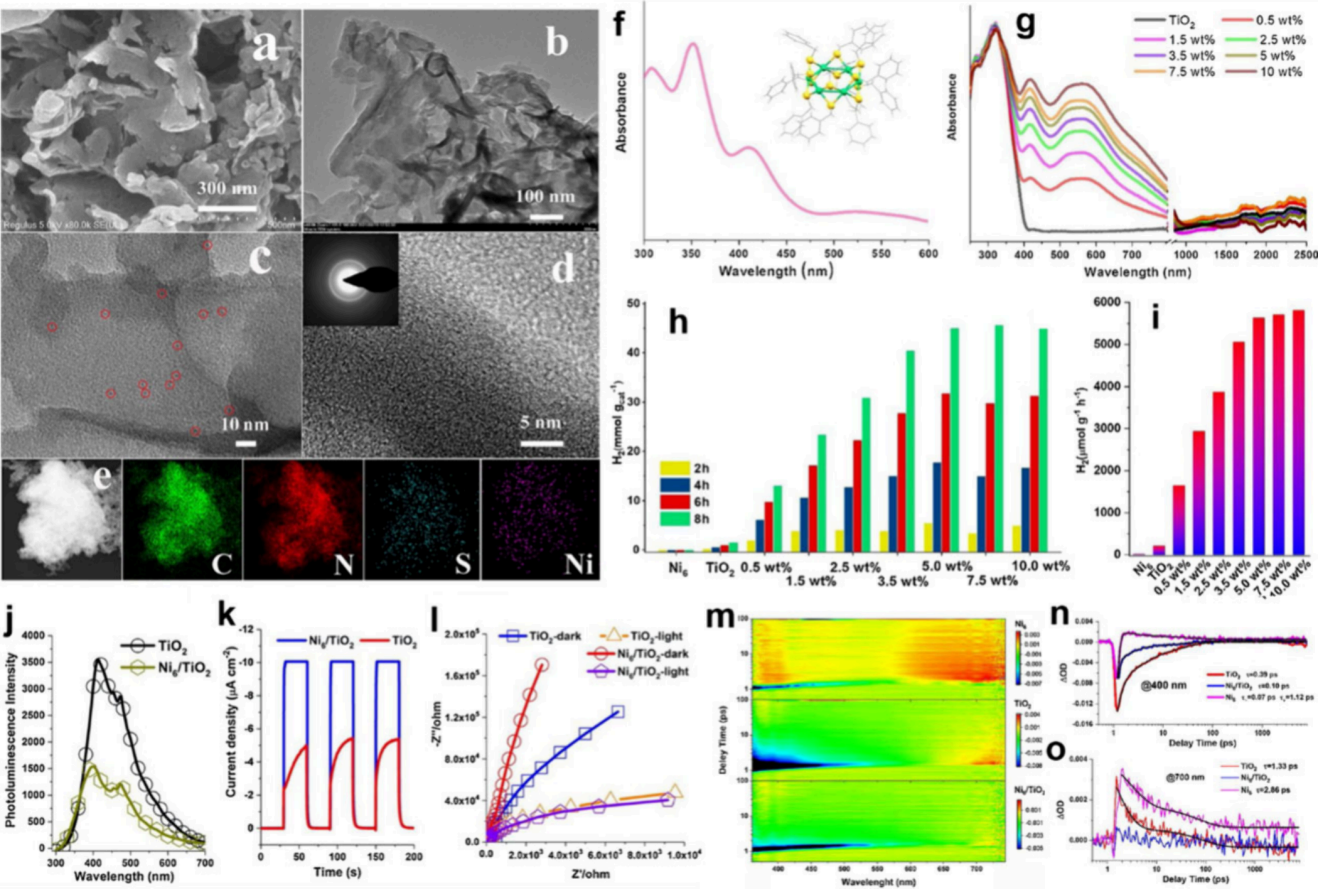
Photos of Ni_6_/g-C_3_N_4_: (a) SEM, (b) TEM, (c) high-magnification TEM, (d) HRTEM, and (e) element mapping. (f) Ni_6_(PMT)_12_ absorption spectrum. Reproduced with permission from ref (10). Copyright 2023, Elsevier B.V. (g) UV/vis diffuse reflection spectra of TiO_2_ before and after the Ni_6_(PMT)_12_ coating. Reproduced with permission from ref (11). Copyright 2021, Elsevier B.V. (h,i) Average hydrogen evolution rate over Ni_6_/TiO_2_ in photocatalytic hydrogen evolution. Steady-state photoluminescence spectroscopy (j), transient photocurrent spectroscopy (k), and electrochemical impedance spectroscopy (EIS, l) on TiO_2_ before and after Ni_6_(PMT)_12_ coating. (m) 2D pseudocolor plots to show the dynamics of the TA signal at 400 (n) and 700 nm (o).

Ni_6_ cluster and g-C_3_N_4_ were first fabricated at room temperature, evenly spreading atomically exact Ni_6_ NCs on the g-C_3_N_4_ nanosheet.^91^ The as-prepared 5%-Ni_6_/g-C_3_N_4_ sample’s SEM and low-magnification TEM images are shown in Figures 4a and 4b, respectively. No discernible shape alteration was seen. A TEM picture demonstrated that the Ni_6_ cluster was successfully loaded onto the surface of g-C_3_N_4_; many black patches (ringed in red) were dispersed over the nanosheet surfaces, Figure 4c. No discernible lattice fringes in HRTEM were seen, even though centered rings in the SEAD pattern suggested the polycrystalline nature of 5%-Ni_6_/g-C_3_N_4_, Figure 4d.^11^ The EDS analysis confirmed Ni_6_/g-C_3_N_4_ hybrid’s successful creation, Figure 4e, demonstrating the uniform distributions of S and Ni elements.^92^ A solvent-evaporating rotary evaporator coated the cluster on commercial titanium dioxide of P25. In PXRD, TEM, and EDS mapping, Ni_6_(PMT)_12_ (PMT: phenylmethanethiol) cluster is evenly loaded on TiO_2_ particles without affecting their crystal structure. The pure Ni_6_(PMT)_12_ cluster shows maximal absorption bands at λ = 350, 410, and 540 nm, Figure 4f, as previously observed. UV–vis–NIR DRS spectra reveal that cluster-deposited TiO_2_ nanoparticles absorb more visible light, Figure 4g. Ni_6_(PMT)_12_ cluster is responsible for the nanocomposite’s visual absorbance, which increases with cluster loading. The heterogeneous catalytic activity of Ni_6_/TiO_2_ nanocomposites is measured by hydrogen evolution in a water solution with methanol as a sacrificial reagent under simulated solar light. The cluster’s photocatalytic role is shown by global hydrogen development in cluster-deposited products, Figure 4h. However, photocatalysis reached an inflection point at 5 wt %, perhaps due to Ni_6_(PMT)_12_ monolayer adsorption on TiO_2_. Most studies demonstrated that appropriate cocatalyst loading enhanced catalytic efficiency.^10,93,94^ According to the EIS Nyquist plot, the Ni_6_/TiO_2_ nanocomposite displays a much bigger arc than TiO_2_ under the conditions (Figure 4l), which suggests that the material’s conductivity will decrease when the nanocluster processes dielectric unignorable ligand groups. The EIS arc of Ni_6_/TiO_2_ as measured is similar to that of pure TiO_2_ under identical circumstances. The movement of electron and hole pairs between nanoclusters and TiO_2_ nanoparticles is, therefore, shown to be significantly influenced by light.

### 3.2 Thermal Catalysis

**Figure 5:**
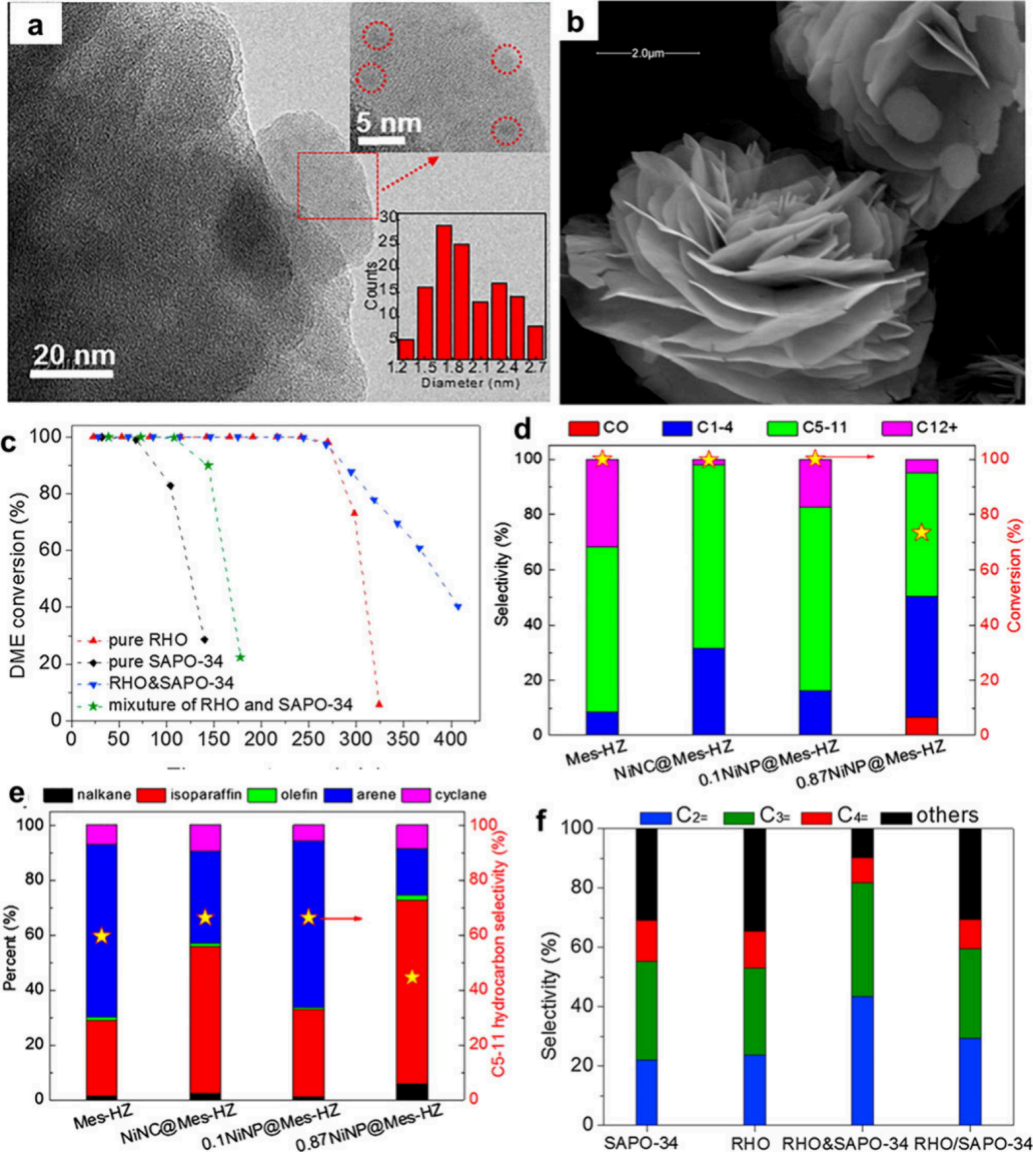
(a) TEM and PSD images of the NiNC@Mes-HZ catalyst. Reproduced with permission from ref (97). Copyright 2018, Elsevier B.V. (b) SEM image of RHO&SAPO-34. (c) DME conversion. (d,e,f) Catalytic activity and product distribution. Reproduced with permission from ref (99). Copyright 2019, Elsevier B.V.

Ni clusters equally distributed 1.2–2.7 nm in the permeable zeolite tube are seen in NiNC@Mes-HZ catalyst, Figure 5a. The lack of sulfur and carbon components implies that the organic thiolate is removed during the 550 °C annealing. Like NiNP@Mes-HZ, Mes-HZSM-5 zeolite was impregnated with Ni(NO_3_)_2_ salt. Ni was largely zeolite framework compensatory cations and some metal oxide.^95^ Nickel oxide dominates meso-zeolite with zeolite metal concentration.^96^ At 20 and 87 nm, Ni particles of 0.1NiNP@Mes-HZ and 0.87NiNP@Mes-HZ are stacked and randomly distributed over the zeolite. Since each nickel atom connects to four sulfur atoms without leaving a portion of surface Ni atoms uncoordinated, the low-atomicity Ni_6_(SR)_12_ cluster was chosen as a model catalyst. The capping sulfur substances may prevent reactants from accessing nickel sites, limiting their catalytic activity. Ni_6_(SR)_12_ clusters had moderate hydrogenation reactivity (<10%) for nitriles, but NH_3_ molecules may give >99% for primary amines.^97^ Silicoaluminaphosphates are 30–40 nm thick and flower-like, Figure 5b.^98^ Catalysis of DTO on molecular sieves was performed at 400 °C with 3,000 mL g^–1^ h^–1^ GHSV. All RHO, RHO&SAPO-34, and SAPO-34 silicoaluminaphosphates converted 100% DME under identical conditions, Figure 5c. The SAPO-34 catalyst has a limited lifespan, losing activity at around 1 h. Although RHO&SAPO-34 and RHO had a longer catalyst lifespan, their catalytic activity declined after 4.5 h. Compared to RHO silicoaluminaphosphates, DME conversion reduced to 5.7% at 5.4 h, whereas RHO&SAPO-34 maintained 40.5% at 6.8 h. It suggests that RHO&SAPO-34’s strong-acid active sites dissociate C5+ products.^99^ Under similar reaction conditions, the 0.11NiNC@Mes-HZ, 0.1NiNP@Mes-HZ, 0.87NiNP@Mes-HZ, and HZSM-5 catalysts performed differently, as shown in Figures 5d,e. 0.11NiNC@Mes-HZ, 0.1NiNP@Mes-HZ, and HZSM-5 catalysts converted 100% DME. Over 0.11NiNC@Mes-HZ catalyst, gasoline hydrocarbon (C5–11) selectivity was 66.4C%, greater than HZSM-5 (59.6%), Figures 5d–f. The selectivity for C12+ hydrocarbons (primarily polymethylarene, as carbon deposition precursors) over 0.11NiNC@Mes-HZ is only 2.0 C%, compared to 31.6 C% over HZSM-5 catalyst.^97^

At a reaction time of 68 min on stream, the DTO process used SAPO-34 molecular sieve catalysts, with ethylene making up 22.1% of the product, propylene 33.3%, and butylene 13.6%. Relative selectivity (C_2–4_) was 69.0%, Figure 5f. A wide variety of products rely on ethylene and propene, two very important raw materials in the petrochemical industry. C_2–4=_ selectivity is 65.5% while ethylene, propylene, and butylene selectivity are all 23.7% for pure RHO. The butylene selectivity dropped to 8.5% after RHO&SAPO-34 increased the ethylene selectivity to 43.6% and the propylene selectivity to 38.1%. A much higher selectivity of 90.2% was achieved by C_2–4=_ compared with RHO and SAPO-34. In the dual-cycle mechanism, RHO&SAPO-34’s high strong-acid density may boost the aromatics-based catalytic cycle’s propagation relative to the olefins-based cycle, leading to remarkable ethylene selectivity.^13,100^

### 3.3 Electrocatalysis

#### 3.3.1 Oxygen Evolution Reaction (OER)

**Figure 6:**
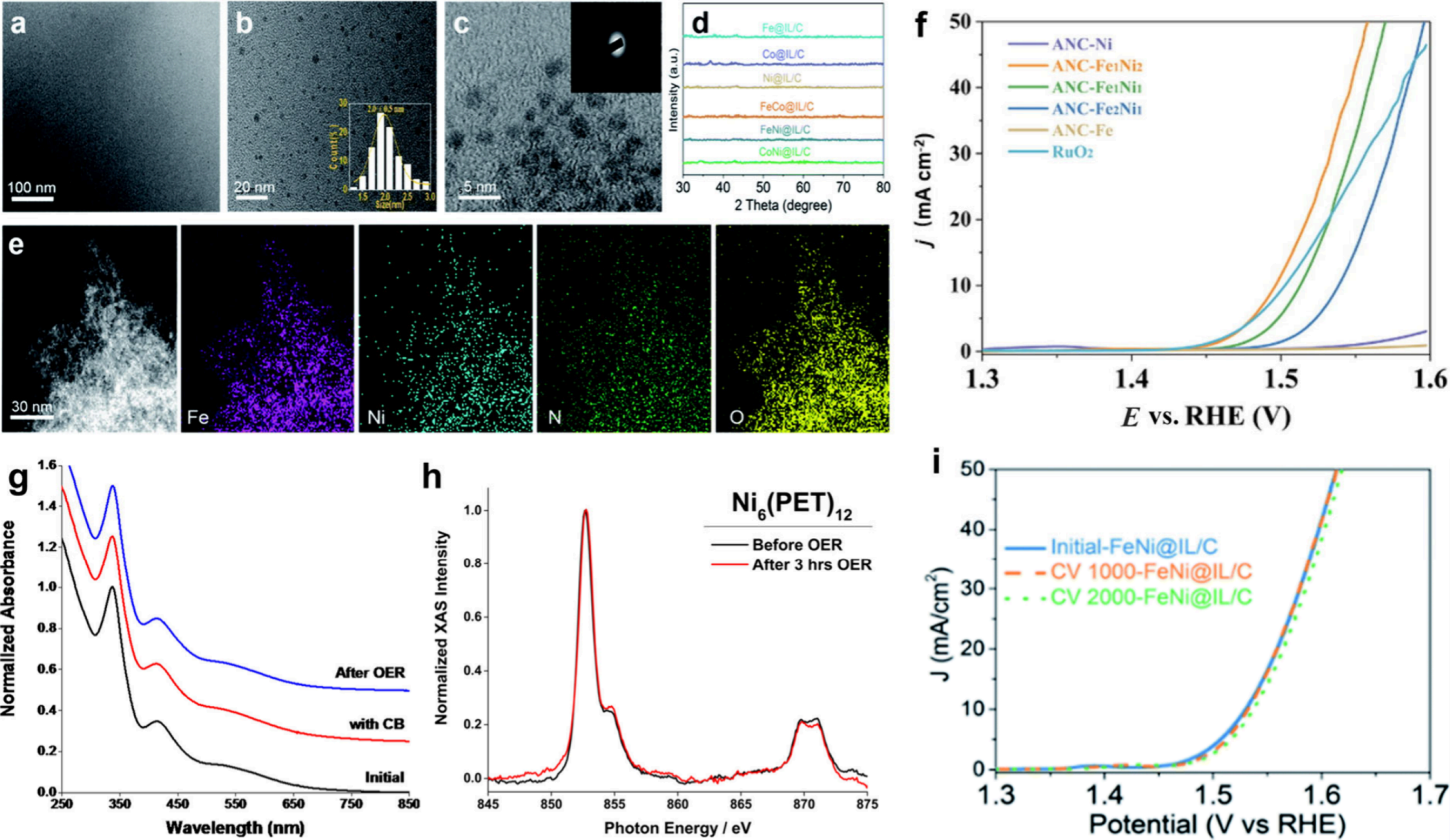
(a–c) HR-TEM images. (d) XRD of M@IL/C. (e) Elemental maps of the FeNi@IL. (f) Assessment of M@IL/C electrocatalytic OER activity in a 1 M KOH solution. Reproduced with permission from ref (101). Copyright 2020, Royal Society of Chemistry. (g) UV–vis and (h) X-ray absorption spectra (XAS) of pristine Ni_6_(PET)_12_ and its post-3-h OER electrolysis. Reproduced with permission from ref (108). Copyright 2016, American Chemical Society. (i) Durability of FeNi@IL/C.

Ultrafine FeNi@IL nanoparticles have a constant diameter of ∼2.0 nm, as shown in Figures 6a,b. The FeNi@IL crystal lattice fringe is not apparent in high-resolution transmission electron microscope (HRTEM) pictures. SAED plot of FeNi@IL in Figure 6c exhibits an amorphous structure.^101^ X-ray diffraction patterns of as-prepared M@IL, where M= Fe, Co, Ni samples show no peaks, indicating their amorphous nature, Figure 6d. FeNi hydroxide’s X-ray diffraction (XRD) pattern exhibits diffraction peaks, proving that 1-aminopropyl-3-methylimidazolium tetrafluoroborate (IL-NH_2_) is essential for amorphous ultrafine hydroxide nanoparticle production. STEM-EDX elemental mapping images of FeNi@IL demonstrate a homogeneous structure with constant Fe, Ni, N, and O distributions, as shown in Figure 6e. N-mapping shows consistent IL-NH_2_ distribution on FeNi hydroxide nanoparticles.^101^

M@IL/C catalysts in OER are investigated using a conventional three-electrode setup and an O_2_-saturated alkaline electrolyte (1 M KOH).^101^ A catalytic activity trend is seen at a 10 mA cm^–2^ potential: FeNi@IL/C < FeCo@IL/C < CoNi < Co < Fe < Ni, (Figure 6f). FeNi@IL/C has a positive start potential at 1.48 V (vs RHE) and a 1.53 V potential at 10 mA cm^–2^, equivalent to commercial RuO_2_ despite lower loading (0.009 mg cm^–2^ vs 0.306 mg cm^–2^). FeNi@IL/C has a lower Tafel slope (54.4 mV dec^–1^) than commercial RuO_2_ (62.5 mV dec^–1^), indicating excellent OER kinetics in assessing catalytic ability using turnover frequency. EIS demonstrates that FeNi@IL/C’s semicircle has a smaller diameter than other catalysts, lowering charge transfer resistance (R_ct_) and increasing electrical conductivity, which boosts the OER activities. CV curves measure the electrochemically active surface area (ECSA), which is positively proportional to Cdl. FeNi@IL/C (2.4 mF cm^–2^) has a greater Cdl than FeCo@IL/C (2.1 mF cm^–2^) and CoNi@IL/C (1.4 mF cm^–2^), supporting reported catalytic activity and EIS. Because these IL–NH_2_-modified ultrafine amorphous bimetallic hydroxide nanoparticles exhibited numerous vacancies, the FeNi@IL/C catalyst has good OER electrocatalytic activity, enhancing active surface area, mass, and charge transport for high reaction kinetics. IL layers on solid supports provide a catalytically active interfacial environment.^101−104^

Numerous UV–vis peaks may be seen in the Ni_6_(PET)_12_ absorbance spectrum due to charge transfer transitions between orbitals centered on S (ligand) and Ni atoms.^90,105^ The S pσ → Ni d transitions have peaks at 337, 416, and 550 nm.^106,107^ Since breaking of Ni–Ni or Ni–S bonds, loss of ligands, or generation of other species like NiO, Ni(OH)_2_, or NiOOH would modify the complex’s absorbance spectra, optical spectroscopy can monitor Ni_6_(PET)_12_ stability.^107^ After 3 h of OER electrolysis and catalytic mixing with CB support, Ni_6_(PET)_12_ optical absorbance spectra are consistent, Figure 6g. To test Ni_6_(PET)_12_ stability, the catalyst was recrystallized after 3 h OER electrolysis and compared pre- and postreaction single crystal unit cells and space groups. These findings show that electrode deposition and anodic electrochemical potentials retained the Ni_6_(PET)_12_ structure. In situ spectroelectrochemistry was employed to detect optical changes produced by deliberate Ni_6_(PET)_12_ degradation at extremely anodic potentials.^108−111^ The stability of Ni_6_(PET)_12_ was confirmed by XAS, which showed very comparable spectra before and after the reaction in Figure 6h. The fact that the peak position and spectral shape were preserved indicates that Ni_6_(PET)_12_ could not have been converted to other Ni species as NiS, NiO, Ni(OH)_2_, NiOOH, during OER electrolysis.^112^ The stability of Ni_6_(PET)_12_ during the electrolysis of the OER is shown by the postreaction crystallographic, XAS, and UV–vis measurements. This chemical stability allowed us to use novel DFT approaches to successfully forecast the OER process at Ni_6_(PET)_12_.^113^ Analyzing electrochemical reactions at catalysts with well-known crystal structures using atomically accurate clusters and molecular catalysts enables atomic-level characterizations of reaction processes, which is challenging with heterogeneous catalyst samples.^114^ Durability, measured by long-term CV scanning, is another important factor in the performance of the OER electrocatalyst performance. FeNi@IL/C catalyst curves and Tafel plots after 1000 and 2000 cycles almost exactly match the initial result, as shown in Figure 6i.^101^

**Figure 7:**
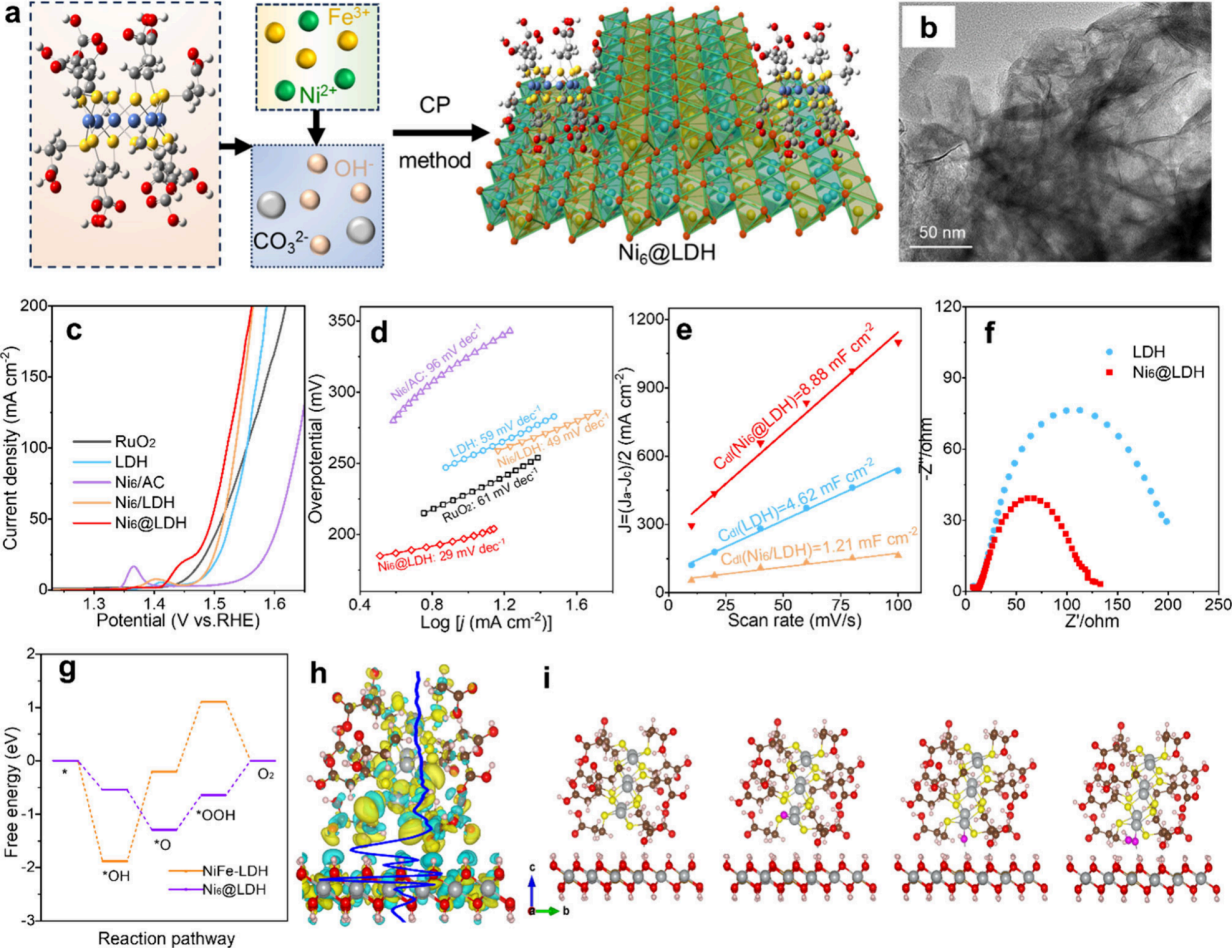
(a) Diagrammatic representation of the steps in creating composites based on the Tiara nickel clusters. Reproduced with permission from ref (12). Copyright 2024, Tsinghua University Press. (b) TEM image. (c) OER polarization curves of the Ni_6_ cluster-based composites in a 1 M KOH electrolyte. (d) Tafel plots. (e) Differences in charging current density displayed against scan rates. (f) Nyquist graphs from EIS. (g) NiFe-LDH and Ni_6_@LDH free energies at a U of 1.23 V. (h) Ni_6_@LDH’s planar-average charge density (blue curve) and charge density differential. (i) NiFe-LDH and Ni_6_@LDH OER setups.

To overcome the difficulties associated with layered double hydroxide (LDH) dispersibility and create the critical structure–property connections required to progress the design of effective OER catalysts, integration techniques that combine monolayer LDHs with atomically precise metal clusters are vital. To facilitate the exfoliation of LDH nanosheets and facilitate effective charge and mass transfer, this has created well-defined Ni_6_@LDH composites in which atomically precise Ni_6_ clusters are evenly distributed and closely integrated with the monodisperse LDHs (thickness ∼0.5–0.8 nm) that have surf-aqua groups.^12^ As seen in Figure 7a, the Ni_6_@NiFe-LDH composites have a synergistic impact on OER at an alkaline condition (1 M KOH electrolyte) in contrast to the simple physical adsorption state (referred to as Ni_6_/AC).^108^

Next, using TEM, the structure and thickness of ultrathin NiFe-LDH monolayers were ascertained (Figure 7b). Under the same circumstances, the most advanced commercial RuO_2_ was also examined for comparison. The OER activities of these catalysts were examined by using linear sweep voltammograms (LSVs). In comparison to the simple physical adsorption Ni_6_/NiFe-LDH counterpart (252 mV@10 mA cm^–2^) and the RuO_2_ (229 mV@10 mA cm^–2^) and NiFe-LDH support alone (255 mV@10 mA cm^–2^), Ni_6_@NiFe-LDH demonstrated the lowest potentials at 198 and 290 mV at a current density of 10 and 100 mA cm^–2^, respectively as shown in Figure 7c. The redox cycle of the Ni^2+^ and Ni^3+^ pair in the electrochemically accessible NiFe-LDH and Ni_6_ clusters is responsible for the notable redox reactions seen in the LDH-based composite.^115^

In general, the alkaline OER process over metal (M) catalysts is supposed to be four consecutive steps (i–iv): i, M + H_2_O ↔ M–OH_ads_ + H^+^ + e^–^; ii, M–OH_ads_ ↔ M–O_ads_ + H^+^ + e^–^; iii, M–O_ads_ + H_2_O ↔ M–OOH_ads_ + H^+^ + e^–^; iv, M–OOH_ads_ ↔ M + O_2(g)_ + H^+^ + e^–^. The rate-determining step (RDS) in the alkaline OER process may be determined by the value of the Tafel slope.^116,117^ The RDS should be the first OH_ads_ formation (i), M–OH_ads_ deprotonation to M–O_ads_ (ii), and M–OOH_ad_s production (iii), according to the Tafel slopes of >120 mV dec^–1^, ∼60 mV dec^–1^, and <40 mV dec^–1^, respectively. Similar Tafel slopes of 59 and 61 mV dec^–1^ (Figure 7d) are shown by both the bare NiFe-LDH and RuO_2_, suggesting that the M–O_ads_ production (step ii) is regarded as the RDS. Additionally, the electrochemically active surface area (ECSA) was calculated using the double-layer capacitance (Cdl). By the observed electrocatalytic activity and EIS, Figure 7e demonstrates that the Cdl of the Ni_6_@NiFe-LDH composite (8.88 mF cm^–2^) is much bigger than that of NiFe-LDH (4.62 mF cm^–2^) and Ni_6_/NiFe-LDH (1.21 mF cm^–2^). Specifically, the smaller semicircle diameter for Ni_6_@NiFe-LDH (Figure 7f) suggests a lower charge transfer resistance (Rct), higher electrical conductivity, and better electrochemical activity when compared to NiFe-LDH alone.^118,119^ The low performance of the Ni_6_/NiFe-LDH catalyst produced by physical adsorption may be attributed to the weak contact between Ni_6_ clusters and NiFe-LDH as well as the need for Ni_6_ clusters to be anchored on the active and defect sites of NiFe-LDH supports. The authors performed systematic DFT calculations to have a better understanding of the reaction mechanism of NiFe-LDH and Ni_6_@NiFe-LDH.^120^ They examined 16 distinct NiFe-LDH combinations to fully assess the model’s logic and chose the model with the lowest relative energy for further computations. As a result, as seen in Figure 7g, the OER free energy shows that OER activity significantly increases when the Ni_6_ cluster forms a composite material with LDH. Remarkably, the production of O* (step ii) with an overpotential of 2.08 V at U = 1.23 V is the RDS for OER in NiFe-LDH. On the other hand, with an overpotential of 0.66 V, the RDS for OER in Ni_6_@LDH is the production of OOH* (step iii).

These findings are in good agreement with the Tafel slopes study. Furthermore, Figure 7i displays the OER reaction configurations for NiFe-LDH and Ni_6_@NiFe-LDH. The work offers a simple and quick method for creating highly distributed Ni_6_@LDHs catalysts by electrostatic ligand-support interaction. Through bonds created by the deprotonated carboxylic acid groups of surface MPA ligands and Ni/Fe cations, the nickel clusters, Ni_6_(MPA)_12_, are integrated with the NiFe-LDH support. In contrast to NiFe-LDH (RDS: M–O_ads_ formation), experimental evidence and mechanistic analysis indicate that the M–OOH_ads_ formation should be the rate-determining step for Ni_6_-based catalysts. Moreover, another system of Ni_6_@CoFe-LDH composites also exhibits a synergistic effect. To create precise structure–activity relationships for the design and optimization of effective OER catalysts, we hope that the combined experimental-computational method may be applied to additional well-defined catalytic systems.^12^

#### 3.3.2 Nitrogen Reduction Reaction (NRR)

**Figure 8:**
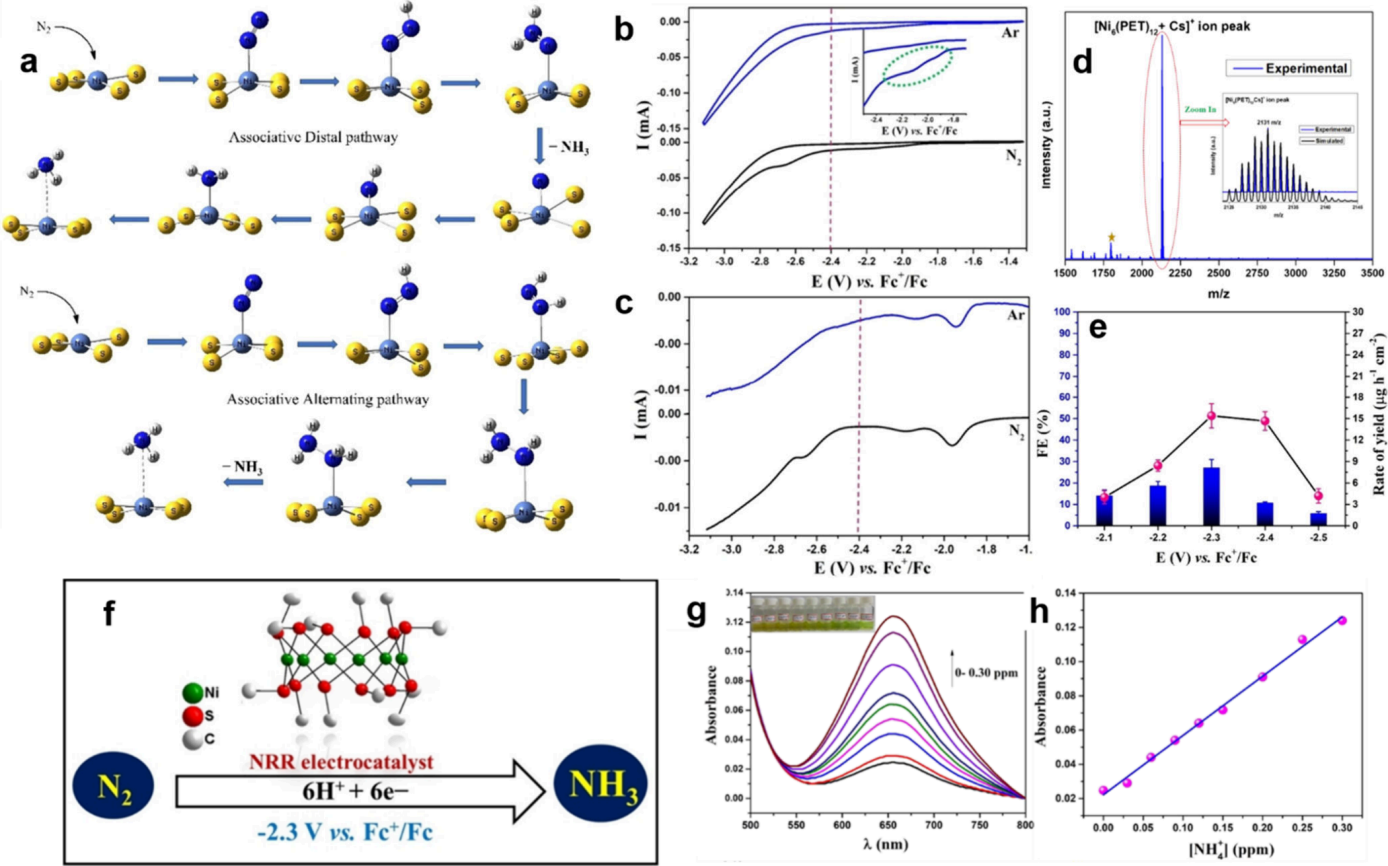
(a) Ni_6_(PET)_12_ electrocatalyst nitrogen reduction mechanism schematic. (b) CV and (c) DPV of [Ni_6_(PET)_12_] in a THF solution. (d) Ni_6_(PET)_12_ ESI-MS data shows the compound’s mass [Ni_6_(PET)_12_+Cs]^+^ 2131 *m*/*z*. (e) IC technique FE and NH_3_ generation rate at various potentials. (f) NRR electrocatalyst [Ni_6_(PET)_12_] scheme. (g) NH_4_^+^ absorption spectra (0–0.30 ppm). (h) Calibration plot. Reproduced with permission from ref (64). Copyright 2023, John Wiley & Sons.

The use of DFT with an implicit solvation model in a THF solvent was used to explain the electrochemical NRR on a Ni_6_(PET)_12_ catalyst in Figure 8a. C, S, and Ni atoms may attach N_2_ to the Ni_6_(PET)_12_ catalyst. The Ni site is the most effective in binding N_2_ with an adsorption energy of −0.48 eV, making it the active site for NRR. The density of states was employed to illustrate the N_2_ molecule-Ni_6_(PET)_12_ catalyst bonding. This work shows that molecular N_2_ adsorbed on the Ni active site of the Ni_6_(PET)_12_ catalyst promotes NRR. Electrochemical reduction of N_2_ to NH_3_ may occur via associative or dissociative routes.^121^ The associative mechanism involves binding of the N_2_ molecule to the Ni_6_(PET)_12_ catalyst. The catalyst-bound N_2_ undergoes protonation to create N_2_H, which dissociates the N–N bond and forms NH_3_. In the dissociative mechanism, the N_2_ molecule splits into two nitrogen atoms over the Ni_6_(PET)_12_ catalyst to generate an N-bonded catalyst. Three proton–electron pairs combine with catalyst-bound N atoms to create NH_3_.^122^ Due to its high kinetic barrier, the dissociation route is often unpopular. Moreover, the significant N≡N bond strength in molecular N_2_ makes it thermodynamically disfavored.^123^ We solely evaluated the associative pathways in this investigation. Although literature reports distal, alternating, and enzymatic associative processes, we studied distal and alternating routes because N_2_ weakly binds to the Ni_6_(PET)_12_ catalyst via Ni active site.^64,124^

CV and DPV analysis of 0.2 mM Ni_6_(PET)_12_ cluster in saturated Ar and N_2_ atmosphere in THF with TBAPF_6_ (tetrabutylammonium hexafluorophosphate) solutions revealed peaks at −1.95 V and −2.20 V vs Fc^+^/Fc. The Ni^2+^/Ni^+^ redox reaction occurs in two crystallographic settings (Figures 8b,c). This matches the crystal structure, where three crystallographically separate Ni centers, two of which are quite near and one dissimilar in bonding and connection. Ni–S bond lengths are 2.18–2.20 Å for Ni(1) and Ni(2), and 2.17–2.23 Å for Ni(3) center. S–Ni–S bond angles vary from 81.5 to 98.6° for Ni(1) and Ni(2) centers and from 81.5 to 99.5° for the Ni(3) core. It found a large peak with an onset potential of about −2.5 V in the spectrum, perhaps caused by the HER. The Ni_6_(PET)_12_ CV in N_2_ atmosphere is similar to that in Ar atmosphere, except for the peak at −2.6 V caused by N_2_ reductive adsorption. The nickel-thiolate cluster was structurally characterized by using ESI-MS and single-crystal X-ray diffraction. ESI-MS spectra showed a high signal at 2131 *m*/*z* for [Ni_6_(PET)_12_Cs]^+^ (Figure 8d). The cluster isotopic pattern matches the simulation precisely.^125^ The computed FE and NH_3_ production rates are shown in Figure 8e. The computed FE and NH_3_ production rates were 25.0% ± 1.7 and 16.2 μg h^–1^ cm^–2^ at −2.3 V vs Fc^+^/Fc. Indophenol yields similar results (Figure 8g,h). Ni_6_(PET)_12_ performs similarly to other Ni-based nanostructures and clusters electrocatalysts for NH_3_ generation.^126^

## 4 Prospects for the Future

The combined knowledge from theoretical and experimental studies on nickel clusters points to several exciting avenues for further investigation and use in the future. These viewpoints center on expanding our knowledge, improving the catalytic performance, and investigating novel applications for nickel clusters.

### 4.1 Proficient Theoretical Understanding

(i) High-Throughput DFT Calculations: To screen a wide variety of nickel clusters with varying sizes, forms, and compositions, future research should make use of high-throughput DFT calculations. Through the identification of ideal structures for certain catalytic processes, high-performance catalysts may be logically designed.
(ii) Machine Learning Integration: Nickel cluster characteristics and catalytic activity may be predicted more quickly by combining machine learning methods with DFT. This integration may provide a potent tool for the discovery of new catalysts based on nickel that have specific functions.
(iii) Dynamic Simulations: The dynamic behavior of nickel clusters under reaction circumstances may be studied using sophisticated simulations, such as ab initio molecular dynamics (AIMD). A deeper understanding of the reaction mechanisms and stability of nickel clusters during catalytic processes will result from this.

### 4.2 Innovative Experiments

(i) Scalable Synthesis Techniques: It is essential to create synthesis techniques that are both scalable and economical to produce nickel clusters with atomic precision. The goal of future research should be to create nickel clusters with uniform size and high purity by refining current synthesis processes and investigating novel approaches.
(ii) Methods of In-Situ Characterization: By using cutting-edge in situ characterization methods, such as environmental transmission electron microscopy (ETEM) and X-ray absorption spectroscopy (XAS), it is possible to get real-time insights into the structural and electronic alterations of nickel clusters during catalysis. This will improve our comprehension of their catalytic processes and direct the development of catalysts that work better.
(iii) Support Material Exploration: Nickel clusters may have their catalytic efficiency greatly increased by looking into other support materials, such as conductive polymers, metal–organic frameworks (MOFs), and 2D materials (e.g., layered double hydroxides).^12^ By enhancing the dispersion, stability, and electrical characteristics of nickel clusters, these supports may produce more effective catalysts.

### 4.3 Utilizing Emerging Domains

(i) Renewable Energy Technologies: Nickel clusters have a lot of promise for use in photocatalytic and electrocatalytic hydrogen generation, among other renewable energy applications. Subsequent investigations need to concentrate on incorporating nickel clusters into photoelectrochemical cells and creating hybrid systems that can effectively convert solar energy into hydrogen.
(ii) Environmental Remediation: Further study on the use of nickel clusters in environmental remediation, namely in the breakdown of contaminants and CO_2_ reduction, is very promising. Investigating nickel clusters’ catalytic qualities for various uses may advance sustainable and environmentally friendly technology.
(iii) Electrochemical Sensors: Nickel clusters may be used to create very selective and sensitive electrochemical sensors that can identify biomolecules and environmental contaminants. Future research should focus on designing nickel cluster-based electrodes logically to maximize the sensitivity and selectivity of these sensors.
(iv) Industrial Catalysis: Increasing the use of nickel clusters in industrial catalytic processes, such as the manufacturing of fine chemicals, ammonia synthesis, and hydrocarbon reforming, may result in more sustainable and effective industrial operations. The commercialization of nickel cluster-based catalysts may be accelerated by industry-academia collaboration.

The integration of theoretical and experimental methods is crucial for the advancement of nickel cluster research in the future. The intricacies of nickel clusters will continue to be uncovered by cutting-edge computational approaches and creative experimental procedures, opening the door for their use in a variety of industries. Future high-performance and sustainable catalytic systems may be developed with the help of nickel clusters by tackling the obstacles and looking into new areas.

## 5 Conclusion

A thorough analysis of nickel clusters using experimental and DFT methods has provided important new information about the structural, electrical, and catalytic characteristics of these materials. These results demonstrate the exceptional potential of nickel clusters as effective catalysts in a variety of applications such as environmental cleanup, hydrogen synthesis, and renewable energy conversion. By prediction of the stability, reactivity, and electrical behavior of nickel clusters, theoretical research using DFT has allowed for a thorough knowledge of the basic characteristics of the material. Simultaneous with these theoretical predictions, experimental validations have shown the practical feasibility of these clusters in catalytic processes. Prospective research directions include combining high-throughput DFT simulations with machine learning methods to evaluate and optimize nickel cluster designs quickly for particular catalytic applications. Furthermore, to create nickel clusters with exact control over their size, shape, and composition—thereby guaranteeing consistency and repeatability in their catalytic performance—scalable synthesis techniques must be developed. Cutting-edge in situ characterization methods, such as microscopy and operando spectroscopy, will enable the identification of active sites and the clarification of reaction processes by offering real-time insights into the dynamic behavior of nickel clusters under catalytic circumstances. We can fully use the potential of nickel clusters by solving these important issues, which will spur innovations that support environmentally friendly technology and effective catalytic processes. To advance science and realize practical applications of nickel-based catalysts, theoretical predictions and experimental validations working together will be crucial. In the end, nickel cluster research and development will open doors for catalytic advances that will facilitate the shift to a more sustainable and energy-efficient future.

## Data Availability

All data supporting the findings of this study are included within the article.

## References

[ref1] Alonso J. Electronic and atomic structure, and magnetism of transition-metal clusters. Chem. Rev. 2000, 100, 637 10.1021/cr980391o. 11749247

[ref2] Shi Q.; Qin Z.; Sharma S.; Li G. Recent progress in heterogeneous catalysis by atomically and structurally precise metal nanoclusters. Chem. Rec. 2021, 21, 879 10.1002/tcr.202100001. 33704895

[ref3] Fang Q.; Qin Z.; Shi Y.; Liu F.; Barkaoui S.; Abroshan H.; Li G. Au/NiO Composite: A Catalyst for One-Pot Cascade Conversion of Furfural. ACS Appl. Energy Mater. 2019, 2, 2654 10.1021/acsaem.9b00001.

[ref4] Li G.; Jin R. Atomically precise gold nanoclusters as new model catalysts. Acc. Chem. Res. 2013, 46, 1749 10.1021/ar300213z. 23534692

[ref5] Riordan C. G. Catalysis by nickel in its high oxidation state. Science 2015, 347, 1203 10.1126/science.aaa7553. 25766220

[ref6] Li Z.; Xu L.; Babar Z.; Raza A.; Zhang Y.; Gu X.; Miao Y.; Zhao Z.; Li G. Fabrication of MXene-Bi_2_WO_6_ heterojunction by Bi_2_Ti_2_O_7_ hinge for extraordinary LED-light-driven photocatalytic performance. Nano Res. 2024, 17, 4729 10.1007/s12274-023-6408-1.

[ref7] Castleman A. Jr; Khanna S. Clusters, superatoms, and building blocks of new materials. J. Phys. Chem. C 2009, 113, 2664 10.1021/jp806850h.

[ref8] Li Z.; Xie Y.; Gao J.; Zhang X.; Zhang J.; Liu Y.; Li G. Promotional effect on multiple active sites in Fe-based oxygen reduction electrocatalysts for zinc-air battery. J. Mater. Chem. A 2023, 11, 26573 10.1039/D3TA03926A.

[ref9] Pembere A. M.; Cui C.; Anumula R.; Wu H.; An P.; Liang T.; Luo Z. A hexagonal Ni6 cluster protected by 2-phenylethanethiol for catalytic conversion of toluene to benzaldehyde. Phys. Chem. Chem. Phys. 2019, 21, 17933 10.1039/C9CP02964H. 31380877

[ref10] Wei J.; Zhao R.; Luo D.; Lu X.; Dong W.; Huang Y.; Cheng X.; Ni Y. Atomically precise Ni6(SC2H4Ph)12 nanoclusters on graphitic carbon nitride nanosheets for boosting photocatalytic hydrogen evolution. J. Colloid Interface Sci. 2023, 631, 212 10.1016/j.jcis.2022.11.010. 36375301

[ref11] Tian F.; Chen J.; Chen F.; Liu Y.; Xu Y.; Chen R. Boosting hydrogen evolution over Ni6(SCH2Ph)12 nanocluster modified TiO2 via pseudo-Z-scheme interfacial charge transfer. Appl. Catal., B 2021, 292, 120158 10.1016/j.apcatb.2021.120158.

[ref12] Gu X.; Guo S.; Zhang Y.; Zhang J.; Sanwal P.; Xu L.; Zhao Z.; Jin R.; Li G. Boosting oxygen evolution performance over synergistic tiara nickel clusters and thin layered double hydroxides. Nano Res. Energy 2024, 3, e9120134 10.26599/NRE.2024.9120134.

[ref13] Shi Q.; Qin Z.; Yu C.; Waheed A.; Xu H.; Gao Y.; Abroshan H.; Li G. Experimental and mechanistic understanding of photo-oxidation of methanol catalyzed by CuO/TiO2-spindle nanocomposite: Oxygen vacancy engineering. Nano Res. 2020, 13, 939 10.1007/s12274-020-2719-7.

[ref14] Liu B.; Lusk M. T.; Ely J. F. Influence of nickel catalyst geometry on the dissociation barriers of H2 and CH4: Ni13 versus Ni (111). J. Phys. Chem. C 2009, 113, 13715 10.1021/jp9003196.

[ref15] Chen L.; Chen G.; Gong C.; Xing Z.; Li J.; Zhang Y.; Xu G.; Li G.; Peng Y. Unveiling Low-valence Platinum Single Atoms in Sulfur-Containing Covalent Organic Frameworks for Exceptional Photocatalytic Hydrogen Evolution. Nat. Commun. 2024, 15, 10501 10.1038/s41467-024-54959-8. 39627232 PMC11614902

[ref16] Zhang J.; Zhang Y.; Qin Z.; Li Z.; Tong Z.; Zhao Z.; Gascón J. A.; Li G. How Carbene Ligands Transform AuAg Alloy Nanoclusters for Electrocatalytic Urea Synthesis. Angew. Chem. Inter. Ed. 2024, 64, e202420993 10.1002/anie.202420993. 39562294

[ref17] Li Q.; Zhang Y.; Xu L.; Chen H.; Zhang L.; Khalid M. S.; Li Z.; Li Z.; Li G. 0D/1D p-n heterostructured composite of bismuth ferrite perovskite clusters on sodium titanate nanotube arouse exceptional blue-light-driven photooxidation. Appl. Catal., B 2025, 361, 124706 10.1016/j.apcatb.2024.124706.

[ref18] Billas I. M.; Chatelain A.; de Heer W. A. Magnetism from the atom to the bulk in iron, cobalt, and nickel clusters. Science 1994, 265, 1682 10.1126/science.265.5179.1682. 17770895

[ref19] Knickelbein M. B. Nickel clusters: The influence of adsorbed CO on magnetic moments. J. Chem. Phys. 2001, 115, 1983 10.1063/1.1388542.

[ref20] Zhang J.; Busari F. K.; Zhang Y.; Guo S.; Zhao Y.; Wang B.; Zeng Q.; Zhao Z.; Li G. RuO_x_ Clusters Anchored on Self-Assembled SnO_2_ Cubic Nanocage for Boosting Sustainable Acidic Water Oxidation. Nano Res. Energy 2024, 3, e9120140 10.26599/NRE.2024.9120140.

[ref21] Zheng K.; Zhang J.; Zhao D.; Yang Y.; Li Z.; Li G. Motif-mediated Au25(SPh)5(PPh3)10 × 2 nanorods with conjugated electron delocalization. Nano Res. 2019, 12, 501 10.1007/s12274-018-2147-0.

[ref22] Barkaoui S.; Wang Y.; Zhang Y.; Gu X.; Li Z.; Wang B.; Baiker A.; Li G.; Zhao Z. Critical Role of NiO Support Morphology for High Activity of Au/NiO Nanocatalysts in CO Oxidation. Iscience 2024, 27, 110255 10.1016/j.isci.2024.110255. 39021794 PMC11253512

[ref23] Qin Z.; Zhao D.; Zhao L.; Xiao Q.; Wu T.; Zhang J.; Wan C.; Li G. Tailoring the stability, photocatalysis and photoluminescence properties of Au 11 nanoclusters via doping engineering. Nanoscale Adv. 2019, 1, 2529 10.1039/C9NA00234K. 36132741 PMC9417908

[ref24] Zheng W.; Zhang J.; Ge Q.; Xu H.; Li W. Effects of CeO2 addition on Ni/Al2O3 catalysts for the reaction of ammonia decomposition to hydrogen. Appl. Catal., B 2008, 80, 98 10.1016/j.apcatb.2007.11.008.

[ref25] Duan X.; Ji J.; Qian G.; Fan C.; Zhu Y.; Zhou X.; Chen D.; Yuan W. Ammonia decomposition on Fe(110), Co(111) and Ni(111) surfaces: A density functional theory study. J. Mole. Catal. A 2012, 357, 81 10.1016/j.molcata.2012.01.023.

[ref26] Kratzer P.; Hammer B.; Norskov J. A theoretical study of CH4 dissociation on pure and gold-alloyed Ni(111) surfaces. J. Chem. Phys. 1996, 105, 5595 10.1063/1.472399.

[ref27] Maroni P.; Papageorgopoulos D. C.; Sacchi M.; Dang T. T.; Beck R. D.; Rizzo T. R. State-Resolved Gas-Surface Reactivity of Methane<? format?> in the Symmetric CH Stretch Vibration on Ni(100). Phys. Rev. Lett. 2005, 94, 246104 10.1103/PhysRevLett.94.246104.

[ref28] Henkelman G.; Arnaldsson A.; Jónsson H. Theoretical calculations of CH4 and H2 associative desorption from Ni (111): Could subsurface hydrogen play an important role?. J. Chem. Phys. 2006, 124, 044706 10.1063/1.2161193. 16460199

[ref29] Nørskov J. K.; Bligaard T.; Logadottir A.; Bahn S.; Hansen L. B.; Bollinger M.; Bengaard H.; Hammer B.; Sljivancanin Z.; Mavrikakis M.; et al. Universality in heterogeneous catalysis. J. Catal. 2002, 209, 275 10.1006/jcat.2002.3615.

[ref30] Wang Y.; Jiang Q.; Xu L.; Han Z.; Guo S.; Li G.; Baiker A. Effect of the configuration of copper oxide-ceria catalysts in NO reduction with CO: superior performance of a copper-ceria solid solution. ACS Appl. Mater. Interfaces 2021, 13, 61078 10.1021/acsami.1c17807. 34905687

[ref31] Zeng Z.-H.; Da Silva J. L.; Li W.-X. Theory of nitride oxide adsorption on transition metal (111) surfaces: a first-principles investigation. Phys. Chem. Chem. Phys. 2010, 12, 2459 10.1039/b920857g. 20449360

[ref32] Zhang Y.; Gu X.; Busari F. K.; Barkaoui S.; Han Z.; Baiker A.; Zhao Z.; Li G. Size hierarchy of gold clusters in nanogold-catalyzed acetylene hydrochlorination. Nano Res. 2024, 17, 9594 10.1007/s12274-024-6976-8.

[ref33] Li Z.; Wang H.; Xie Y.; Zhang Y.; Gao J.; Xu Y.; Chen L.; Xu L.; Wang E.; Li G. MoP Assists the Promotion on Fe2P and FeN4 for Oxygen Reduction and Zinc-Air Battery. J. Colloid Interface Sci. 2025, 681, 16 10.1016/j.jcis.2024.11.150. 39581074

[ref34] Shi Y.; Tian S.; Shi Q.; Zhang Y.; Waheed A.; Cao Y.; Li G. Cascade Aldol Condensation of Aldehyde via the Aerobic Oxidation of Ethanol over Au/NiO Composite. Nanoscale Adv. 2019, 1, 3654 10.1039/C9NA00412B. 36133540 PMC9418894

[ref35] Gawande M. B.; Fornasiero P.; Zbořil R. Carbon-based single-atom catalysts for advanced applications. ACS Catal. 2020, 10, 2231 10.1021/acscatal.9b04217.

[ref36] Cao Y.; Su Y.; Xu L.; Yang X.; Han Z.; Cao R.; Li G. Oxygen vacancy-rich amorphous FeNi hydroxide nanoclusters as an efficient electrocatalyst for water oxidation. J. Energy Chem. 2022, 71, 167 10.1016/j.jechem.2022.03.044.

[ref37] Chen Y.; Liu C.; Abroshan H.; Li Z.; Wang J.; Li G.; Haruta M. Phosphine/phenylacetylide-ligated Au clusters for multicomponent coupling reactions. J. Catal. 2016, 340, 287 10.1016/j.jcat.2016.05.023.

[ref38] Miao M.; Gong X.; Lei S.; Wang L.; Sha M.; Meng Q. The graphene-supported non-noble metal catalysts activate ammonia decomposition: A DFT study. Chem. Phys. 2021, 548, 111249 10.1016/j.chemphys.2021.111249.

[ref39] Shi Q.; Zhang X.; Liu X.; Xu L.; Liu B.; Zhang J.; Xu H.; Han Z.; Li G. In-situ exfoliation and assembly of 2D/2D g-C3N4/TiO2 (B) hierarchical microflower: Enhanced photo-oxidation of benzyl alcohol under visible light. Carbon 2022, 196, 401 10.1016/j.carbon.2022.05.007.

[ref40] Li L.; Huang R.; Cao X.; Wen Y. Computational screening of efficient graphene-supported transition metal single atom catalysts toward the oxygen reduction reaction. J. Mater. Chem. A 2020, 8, 19319 10.1039/D0TA06892F.

[ref41] Liu Y.; Hua Y.; Jiang M.; Jiang G.; Chen J. Theoretical study of the geometries and dissociation energies of molecular water on neutral aluminum clusters Aln (n= 2–25). J. Chem. Phys. 2012, 136, 084703 10.1063/1.3685603. 22380055

[ref42] López Arvizu G.; Calaminici P. Assessment of density functional theory optimized basis sets for gradient corrected functionals to transition metal systems: the case of small Nin (n⩽ 5) clusters. J. Chem. Phys. 2007, 126, 194102 10.1063/1.2735311. 17523793

[ref43] Ma L.; Wang J.; Hao Y.; Wang G. Density functional theory study of FePdn (n= 2–14) clusters and interactions with small molecules. Comput. Mater. Sci. 2013, 68, 166 10.1016/j.commatsci.2012.10.014.

[ref44] Parks E.; Zhu L.; Ho J.; Riley S. The structure of small nickel clusters. I. Ni3-Ni15. J. Chem. Phys. 1994, 100, 7206–7222. 10.1063/1.466868.

[ref45] Farmanzadeh D.; Abdollahi T. Investigation on the chemical active sites of copper nanoclusters as nanocatalyst for the adsorption of acetylene: calibration of DFT method and basis set. Theor. Chem. Acc. 2016, 135, 1 10.1007/s00214-016-1806-z.

[ref46] Kung H. H.; Kung M. C. Nanotechnology: applications and potentials for heterogeneous catalysis. Catal. Today 2004, 97, 219 10.1016/j.cattod.2004.07.055.

[ref47] Lewis N. S.; Nocera D. G. Powering the planet: Chemical challenges in solar energy utilization. Proc. Nat. Acad. Sci. 2006, 103, 15729 10.1073/pnas.0603395103. 17043226 PMC1635072

[ref48] Du P.; Eisenberg R. Catalysts made of earth-abundant elements (Co, Ni, Fe) for water splitting: recent progress and future challenges. Energy Environ. Sci. 2012, 5, 6012 10.1039/c2ee03250c.

[ref49] Cline E. D.; Adamson S. E.; Bernhard S. Homogeneous catalytic system for photoinduced hydrogen production utilizing iridium and rhodium complexes. Inorg. Chem. 2008, 47, 10378 10.1021/ic800988b. 18939819

[ref50] Gallon B. J.; Kojima R. W.; Kaner R. B.; Diaconescu P. L. Palladium nanoparticles supported on polyaniline nanofibers as a semi-heterogeneous catalyst in water. Angew. Chem., Int. Ed. 2007, 46, 7251 10.1002/anie.200701389. 17657750

[ref51] Ivanov S. A.; Kozee M. A.; Merrill W. A.; Agarwal S.; Dahl L. F. Cyclo-[Ni (μ 2-SPh) 2] 9 and cyclo-[Ni (μ 2-SPh) 2] 11: new oligomeric types of toroidal nickel (ii) thiolates containing geometrically unprecedented 9-and 11-membered ring systems. J. Chem. Soc., Dalton Trans. 2002, 4105 10.1039/B204273H.

[ref52] Lowry M. S.; Hudson W. R.; Pascal R. A.; Bernhard S. Accelerated luminophore discovery through combinatorial synthesis. J. Am. Chem. Soc. 2004, 126, 14129 10.1021/ja047156+. 15506778

[ref53] QIAN H.; BARRY E.; ZHU Y.; JIN R. Doping 25-atom and 38-atom gold nanoclusters with palladium. Acta Phys. Chim. Sinica 2011, 27, 513 10.3866/PKU.WHXB20110304.

[ref54] Yang Z.; Smetana A. B.; Sorensen C. M.; Klabunde K. J. Synthesis and characterization of a new tiara Pd(II) thiolate complex,[Pd (SC12H25) 2] 6, and its solution-phase thermolysis to prepare nearly monodisperse palladium sulfide nanoparticles. Inorg. Chem. 2007, 46, 2427 10.1021/ic061242o. 17335274

[ref55] Du X. L.; Wang X. L.; Li Y. H.; Wang Y. L.; Zhao J. J.; Fang L. J.; Zheng L. R.; Tong H.; Yang H. G. Isolation of single Pt atoms in a silver cluster: forming highly efficient silver-based cocatalysts for photocatalytic hydrogen evolution. Chem. Commun. 2017, 53, 9402 10.1039/C7CC04061J. 28677696

[ref56] Tian K.; Liu W.-J.; Jiang H. Comparative investigation on photoreactivity and mechanism of biogenic and chemosythetic Ag/C3N4 composites under visible light irradiation. ACS Sust. Chem. Engin. 2015, 3, 269 10.1021/sc500646a.

[ref57] Cai H.; Wang B.; Xiong L.; Bi J.; Hao H.; Yu X.; Li C.; Liu J.; Yang S. Boosting photocatalytic hydrogen evolution of gC 3 N 4 catalyst via lowering the Fermi level of co-catalyst. Nano Res. 2022, 15, 1128 10.1007/s12274-021-3615-5.

[ref58] Xiao X.; Lin S.; Zhang L.; Meng H.; Zhou J.; Li Q.; Liu J.; Qiao P.; Jiang B.; Fu H. Constructing Pd-N interactions in Pd/gC 3 N 4 to improve the charge dynamics for efficient photocatalytic hydrogen evolution. Nano Res. 2022, 15, 2928 10.1007/s12274-021-3905-y.

[ref59] Uekert T.; Kasap H.; Reisner E. Photoreforming of nonrecyclable plastic waste over a carbon nitride/nickel phosphide catalyst. J. Am. Chem. Soc. 2019, 141, 15201 10.1021/jacs.9b06872. 31462034 PMC7007225

[ref60] Yin Q.; Tan J. M.; Besson C.; Geletii Y. V.; Musaev D. G.; Kuznetsov A. E.; Luo Z.; Hardcastle K. I.; Hill C. L. A Fast Soluble Carbon-Free Molecular Water Oxidation Catalyst Based on Abundant Metals. Science 2010, 328, 342 10.1126/science.1185372. 20223949

[ref61] Zhang J.; Xu L.; Yang X.; Guo S.; Zhang Y.; Zhao Y.; Wu G.; Li G. Amorphous MnRuOx Containing Microcrystalline for Enhanced Acidic Oxygen-Evolution Activity and Stability. Angew. Chem., Int. Ed. 2024, 63, e202405641 10.1002/anie.202405641. 38818616

[ref62] Zhang J.; Zhou W.; Zhao J.; Xu L.; Jiang X.; Li Z.; Peng Y.; Li G. Intrareticular Exciton Effects Regulate Photocatalytic Activity in Donor-Acceptor Olefin-Linked Covalent Organic Frameworks. Small 2024, 20, 2408324 10.1002/smll.202408324. 39491491

[ref63] Jin R.; Zeng C.; Zhou M.; Chen Y. Atomically precise colloidal metal nanoclusters and nanoparticles: fundamentals and opportunities. Chem. Rev. 2016, 116, 10346 10.1021/acs.chemrev.5b00703. 27585252

[ref64] Maman M. P.; Gurusamy T.; Pal A. K.; Jana R.; Ramanujam K.; Datta A.; Mandal S. Electrocatalytic Reduction of Nitrogen to Ammonia Using Tiara-like Phenylethanethiolated Nickel Cluster. Angew. Chem., Int. Ed. 2023, 135, e202305462 10.1002/ange.202305462. 37129995

[ref65] Sanwal P.; Raza A.; Li G. Unlocking the Potential of Metal Oxide and Hydroxide Clusters: Toward Next-Generation Electrocatalysts for Sustainable Energy Utilizations. Chem. Commun. 2024, 60, 9918. 10.1039/d4cc02722a 39145411

[ref66] Chaves A. S.; Piotrowski M. J.; Da Silva J. L. Evolution of the structural, energetic, and electronic properties of the 3d, 4d, and 5d transition-metal clusters (30 TM n systems for n= 2–15): a density functional theory investigation. Phys. Chem. Chem. Phys. 2017, 19, 15484 10.1039/C7CP02240A. 28580970

[ref67] Parker T. M.; Burns L. A.; Parrish R. M.; Ryno A. G.; Sherrill C. D. Levels of symmetry adapted perturbation theory (SAPT). I. Efficiency and performance for interaction energies. J. Chem. Phys. 2014, 140, 094106 10.1063/1.4867135. 24606352

[ref68] Hohenstein E. G.; Parrish R. M.; Sherrill C. D.; Turney J. M.; Schaefer H. F. Large-scale symmetry-adapted perturbation theory computations via density fitting and Laplace transformation techniques: Investigating the fundamental forces of DNA-intercalator interactions. J. Chem. Phys. 2011, 135, 174107 10.1063/1.3656681. 22070292

[ref69] Weigend F.; Ahlrichs R. Balanced basis sets of split valence, triple zeta valence and quadruple zeta valence quality for H to Rn: Design and assessment of accuracy. Phys. Chem. Chem. Phys. 2005, 7, 3297 10.1039/b508541a. 16240044

[ref70] Da Silva J. L.; Kim H. G.; Piotrowski M. J.; Prieto M. J.; Tremiliosi-Filho G. Reconstruction of core and surface nanoparticles: The example of Pt55 and Au55. Phys. Rev. B 2010, 82, 205424 10.1103/PhysRevB.82.205424.

[ref71] Li G.; Zeng C.; Jin R. Chemoselective hydrogenation of nitrobenzaldehyde to nitrobenzyl alcohol with unsupported Au nanorod catalysts in water. J. Phys. Chem. C 2015, 119, 11143 10.1021/jp511930n.

[ref72] Song W.; Lu W.-C.; Wang C.; Ho K. Magnetic and electronic properties of the nickel clusters Nin (n⩽ 30). Comput. Theor. Chem. 2011, 978, 41 10.1016/j.comptc.2011.09.028.

[ref73] Zhang G.; Wang R.; Li G. Non-metallic gold nanoclusters for oxygen activation and aerobic oxidation. Chin. Chem. Lett. 2018, 29, 687 10.1016/j.cclet.2018.01.043.

[ref74] Zhou S.; Lin S.; Guo H. First-principles insights into ammonia decomposition catalyzed by Ru clusters anchored on carbon nanotubes: size dependence and interfacial effects. J. Phys. Chem. C 2018, 122, 9091 10.1021/acs.jpcc.8b01965.

[ref75] de Amorim R. V.; Batista K. E.; Nagurniak G. R.; Orenha R. P.; Parreira R. L.; Piotrowski M. J. CO, NO, and SO adsorption on Ni nanoclusters: a DFT investigation. Dalton Trans. 2020, 49, 6407 10.1039/D0DT00288G. 32352455

[ref76] Seenivasan H.; Tiwari A. K. Enhancing methane dissociation with nickel nanoclusters. Comput. Theor. Chem. 2015, 1064, 7 10.1016/j.comptc.2015.04.016.

[ref77] Farmanzadeh D.; Abdollahi T. H2 adsorption on free and graphene-supported Ni nanoclusters: a theoretical study. Surf. Sci. 2018, 668, 85 10.1016/j.susc.2017.10.024.

[ref78] Nave S.; Tiwari A. K.; Jackson B. Methane dissociation and adsorption on Ni (111), Pt (111), Ni (100), Pt (100), and Pt (110)-(1× 2): energetic study. J. Chem. Phys. 2010, 132, 054705 10.1063/1.3297885. 20136331

[ref79] Wang S.; Petzold V.; Tripkovic V.; Kleis J.; Howalt J. G.; Skulason E.; Fernández E.; Hvolbæk B.; Jones G.; Toftelund A.; et al. Universal transition state scaling relations for (de) hydrogenation over transition metals. Phys. Chem. Chem. Phys. 2011, 13, 20760 10.1039/c1cp20547a. 21996683

[ref80] Vajda S.; Pellin M. J.; Greeley J. P.; Marshall C. L.; Curtiss L. A.; Ballentine G. A.; Elam J. W.; Catillon-Mucherie S.; Redfern P. C.; Mehmood F.; Zapol P. Subnanometre platinum clusters as highly active and selective catalysts for the oxidative dehydrogenation of propane. Nat. Mater. 2009, 8, 213 10.1038/nmat2384. 19202544

[ref81] Yudanov I. V.; Genest A.; Schauermann S.; Freund H.-J.; Rösch N. Size dependence of the adsorption energy of CO on metal nanoparticles: a DFT search for the minimum value. Nano Lett. 2012, 12, 2134 10.1021/nl300515z. 22468882

[ref82] Calaminici P. Is the trend of the polarizability per atom for all small 3d transition metal clusters the same? The case of Nin (n⩽ 5) clusters. J. Chem. Phys. 2008, 128, 164317 10.1063/1.2909201. 18447449

[ref83] Zhang Y.; Li Z.; Zhang J.; Xu L.; Han Z.; Baiker A.; Li G. Nanostructured Ni-MoC_x_: An Efficient Non-Noble Metal Catalyst for the Chemoselective Hydrogenation of Nitroaromatics. Nano Res. 2023, 16, 8919 10.1007/s12274-023-5598-x.

[ref84] Öström H.; Ogasawara H.; Näslund L.-Å.; Pettersson L.; Nilsson A. Physisorption-induced CH bond elongation in methane. Phys. Rev. Lett. 2006, 96, 146104 10.1103/PhysRevLett.96.146104. 16712100

[ref85] Kagalwala H. N.; Chirdon D. N.; Mills I. N.; Budwal N.; Bernhard S. Light-driven hydrogen generation from microemulsions using metallosurfactant catalysts and oxalic acid. Inorg. Chem. 2017, 56, 10162 10.1021/acs.inorgchem.7b00463. 28488856

[ref86] Pollitt S.; Pittenauer E.; Rameshan C.; Schachinger T.; Safonova O. V.; Truttmann V.; Bera A.; Allmaier G. n.; Barrabés N.; Rupprechter G. n. Synthesis and Properties of Monolayer-Protected Co x (SC2H4Ph) m Nanoclusters. J. Phys. Chem. C 2017, 121, 10948 10.1021/acs.jpcc.6b12076.

[ref87] Shi Q.; Wei X.; Raza A.; Li G. Recent Advances in Aerobic Photo-Oxidation of Methanol to Valuable Chemicals. ChemCatChem 2021, 13, 3381 10.1002/cctc.202100104.

[ref88] Chen L.; Chen G.; Leung C.-F.; Yiu S.-M.; Ko C.-C.; Anxolabéhère-Mallart E.; Robert M.; Lau T.-C. Dual homogeneous and heterogeneous pathways in photo-and electrocatalytic hydrogen evolution with nickel (II) catalysts bearing tetradentate macrocyclic ligands. ACS Catal. 2015, 5, 356 10.1021/cs501534h.

[ref89] Hui-Liang Z.; Wei Z.; Xiang D.; Xian-Cai F. A study of catalytic activity, constituent, and structure of V—Ag catalyst for selective oxidation of toluene to benzaldehyde. J. Catal. 1991, 129, 426 10.1016/0021-9517(91)90046-7.

[ref90] Kagalwala H. N.; Gottlieb E.; Li G.; Li T.; Jin R.; Bernhard S. Photocatalytic hydrogen generation system using a nickel-thiolate hexameric cluster. Inorg. Chem. 2013, 52, 9094 10.1021/ic4013069. 23865570

[ref91] Hojamberdiev M.; Khan M. M.; Kadirova Z.; Kawashima K.; Yubuta K.; Teshima K.; Riedel R.; Hasegawa M. Synergistic effect of g-C3N4, Ni(OH)2 and halloysite in nanocomposite photocatalyst on efficient photocatalytic hydrogen generation. Renew. Energy 2019, 138, 434 10.1016/j.renene.2019.01.103.

[ref92] Han Y. Y.; Lu X. L.; Tang S. F.; Yin X. P.; Wei Z. W.; Lu T. B. Metal-free 2D/2D heterojunction of graphitic carbon nitride/graphdiyne for improving the hole mobility of graphitic carbon nitride. Adv. Energy Mater. 2018, 8, 1702992 10.1002/aenm.201702992.

[ref93] Mouhat F.; Coudert F.-X. Necessary and sufficient elastic stability conditions in various crystal systems. Phys. Rev. B 2014, 90, 224104 10.1103/PhysRevB.90.224104.

[ref94] Zheng Y.; Jiao Y.; Jaroniec M.; Qiao S. Z. Advancing the electrochemistry of the hydrogen-evolution reaction through combining experiment and theory. Angew. Chem., Int. Ed. 2015, 54, 52 10.1002/anie.201407031. 25384712

[ref95] Maia A.; Louis B.; Lam Y.; Pereira M. Ni-ZSM-5 catalysts: Detailed characterization of metal sites for proper catalyst design. J. Catal. 2010, 269, 103 10.1016/j.jcat.2009.10.021.

[ref96] Almutairi S. M.; Mezari B.; Magusin P. C.; Pidko E. A.; Hensen E. J. Structure and reactivity of Zn-modified ZSM-5 zeolites: the importance of clustered cationic Zn complexes. ACS Catal. 2012, 2, 71 10.1021/cs200441e.

[ref97] Wen Z.; Li Z.; Ge Q.; Zhou Y.; Sun J.; Abroshan H.; Li G. Robust nickel cluster@ Mes-HZSM-5 composite nanostructure with enhanced catalytic activity in the DTG reaction. J. Catal. 2018, 363, 26 10.1016/j.jcat.2018.04.010.

[ref98] Chai X.; Li T.; Chen M.; Jin R.; Ding W.; Zhu Y. Suppressing the active site-blocking impact of ligands of Ni6(SR)12 clusters with the assistance of NH3 on catalytic hydrogenation of nitriles. Nanoscale 2018, 10, 19375 10.1039/C8NR03700K. 30307002

[ref99] Wang F.; Wen Z.; Qin Z.; Fang Q.; Ge Q.; Li Z.; Sun J.; Li G. Manganese cluster induce the control synthesis of RHO-and CHA-type silicoaluminaphosphates for dimethylether to light olefin conversion. Fuel 2019, 244, 104 10.1016/j.fuel.2019.02.013.

[ref100] Zhang J.; Raza A.; Zhao Y.; Guo S.; Babar Z.; Xu L.; Cao C.; Li G. Intrinsically Robust Cubic MnCoOx Solid Solution: Achieving High Activity for Sustainable Acidic Water Oxidation. J. Mater. Chem. A 2023, 11, 25345 10.1039/D3TA05233H.

[ref101] Cao Y.; Guo S.; Yu C.; Zhang J.; Pan X.; Li G. Ionic liquid-assisted one-step preparation of ultrafine amorphous metallic hydroxide nanoparticles for the highly efficient oxygen evolution reaction. J. Mater. Chem. A 2020, 8, 15767 10.1039/D0TA00434K.

[ref102] Gao M.; Sheng W.; Zhuang Z.; Fang Q.; Gu S.; Jiang J.; Yan Y. Efficient water oxidation using nanostructured α-nickel-hydroxide as an electrocatalyst. J. Am. Chem. Soc. 2014, 136, 7077 10.1021/ja502128j. 24761994

[ref103] Zhang J.; Xie Y.; Jiang Q.; Guo S.; Huang J.; Xu L.; Wang Y.; Li G. Facile Synthesis of Cobalt Clusters-CoNx Composites: Synergistic Effect Boosts up Electrochemical Oxygen Reduction. J. Mater. Chem. A 2022, 10, 16920 10.1039/D2TA04413G.

[ref104] Li Z.; Xu M.; Wang J.; Zhang Y.; Liu W.; Gu X.; Han Z.; Ye W.; Li G. Boosting up Electrosynthesis of Urea with Nitrate and Carbon Dioxide via Synergistic Effect of Metallic Iron Cluster and Single-Atom. Small 2024, 20, 2400036 10.1002/smll.202400036. 38747043

[ref105] Zhang W.; Hong J.; Zheng J.; Huang Z.; Zhou J.; Xu R. Nickel-thiolate complex catalyst assembled in one step in water for solar H2 production. J. Am. Chem. Soc. 2011, 133, 20680 10.1021/ja208555h. 22133284

[ref106] Gorelsky S. I.; Basumallick L.; Vura-Weis J.; Sarangi R.; Hodgson K. O.; Hedman B.; Fujisawa K.; Solomon E. I. Spectroscopic and DFT Investigation of [M {HB (3, 5-i Pr2pz) 3}(SC6F5)](M= Mn, Fe, Co, Ni, Cu, and Zn) Model Complexes: Periodic Trends in Metal- Thiolate Bonding. Inorg. Chem. 2005, 44, 4947 10.1021/ic050371m. 15998022 PMC2593087

[ref107] John N. S.; Kulkarni G.; Datta A.; Pati S. K.; Komori F.; Kavitha G.; Narayana C.; Sanyal M. Magnetic interactions in layered nickel alkanethiolates. J. Phys. Chem. C 2007, 111, 1868 10.1021/jp0675072.

[ref108] Kauffman D. R.; Alfonso D.; Tafen D. N.; Lekse J.; Wang C.; Deng X.; Lee J.; Jang H.; Lee J.-s.; Kumar S.; Matranga C. Electrocatalytic oxygen evolution with an atomically precise nickel catalyst. ACS Catal. 2016, 6, 1225 10.1021/acscatal.5b02633.

[ref109] Xiao H. L.; Jian F. F.; Zhang K. J. Synthesis and structure analysis of α and β forms of [12] metallacrown-6 nickel (II) complex:[Ni 6 (SCH 2 CH 2 CH 3) 12]. Bull. Korean Chem. Soc. 2009, 30, 846 10.5012/bkcs.2009.30.4.846.

[ref110] Tan C.; Jin M.; Ma X.; Zhu Q.; Huang Y.; Wang Y.; Hu S.; Sheng T.; Wu X. In situ synthesis of nickel tiara-like clusters with two different thiolate bridges. Dalton Trans. 2012, 41, 8472 10.1039/c2dt30524k. 22653469

[ref111] Capdevila M.; González-Duarte P.; Sola J.; Foces-Foces C.; Cano F. H.; Martínez-Ripoll M. Preparation and X-ray crystal structure of [Ni6 {μ-S (CH2) 3N (CH3) 2} 12], a cyclic hexameric homothiolate of nickel. Polyhedron 1989, 8, 1253 10.1016/S0277-5387(00)86522-5.

[ref112] McCrory C. C.; Jung S.; Ferrer I. M.; Chatman S. M.; Peters J. C.; Jaramillo T. F. Benchmarking hydrogen evolving reaction and oxygen evolving reaction electrocatalysts for solar water splitting devices. J. Am. Chem. Soc. 2015, 137, 4347 10.1021/ja510442p. 25668483

[ref113] Lussier A.; Sofie S.; Dvorak J.; Idzerda Y. Mechanism for SOFC anode degradation from hydrogen sulfide exposure. Int. J. Hydrogen Energy 2008, 33, 3945 10.1016/j.ijhydene.2007.11.033.

[ref114] Nakamura M.; Fujimori A.; Sacchi M.; Fuggle J.; Misu A.; Mamori T.; Tamura H.; Matoba M.; Anzai S. Metal-nonmetal transition in NiS induced by Fe and Co substitution: X-ray-absorption spectroscopic study. Phys. Rev. B 1993, 48, 16942 10.1103/PhysRevB.48.16942. 10008293

[ref115] Pan Y.; Chen J.; Gong S.; Wang Z. Co-synthesis of atomically precise nickel nanoclusters and the pseudo-optical gap of Ni 4 (SR) 8. Dalton Trans. 2018, 47, 11097 10.1039/C8DT02059K. 30040107

[ref116] Lyons M. E.; Doyle R. L. Oxygen evolution at oxidised iron electrodes: a tale of two slopes. Int. J. Electrochem. Sci. 2012, 7, 9488 10.1016/S1452-3981(23)16213-4.

[ref117] Doyle R. L.; Godwin I. J.; Brandon M. P.; Lyons M. E. Redox and electrochemical water splitting catalytic properties of hydrated metal oxide modified electrodes. Phys. Chem. Chem. Phys. 2013, 15, 13737 10.1039/c3cp51213d. 23652494

[ref118] Yan D.; Li Y.; Huo J.; Chen R.; Dai L.; Wang S. Defect Chemistry of Nonprecious-Metal Electrocatalysts for Oxygen Reactions. Adv. Mater. 2017, 29, 1606459 10.1002/adma.201606459. 28508469

[ref119] Zhang J.; Wang T.; Liu P.; Liao Z.; Liu S.; Zhuang X.; Chen M.; Zschech E.; Feng X. Efficient hydrogen production on MoNi4 electrocatalysts with fast water dissociation kinetics. Nat. Commun. 2017, 8, 15437 10.1038/ncomms15437. 28513620 PMC5442356

[ref120] Chen Y.; Gu X.; Guo S.; Zhang J.; Barkaoui S.; Xu L.; Li G. Enhancing the Performance of 2D Ni-Fe Layered Double Hydroxides by Cabbage-Inspired Carbon Conjunction for Oxygen Evolution Reactions. ChemSusChem 2024, e202400309 10.1002/cssc.202400309. 38610067

[ref121] Li M.; Huang H.; Low J.; Gao C.; Long R.; Xiong Y. Recent progress on electrocatalyst and photocatalyst design for nitrogen reduction. Small Methods 2019, 3, 1800388 10.1002/smtd.201800388.

[ref122] Liu H.; Wei L.; Liu F.; Pei Z.; Shi J.; Wang Z.-j.; He D.; Chen Y. Homogeneous, heterogeneous, and biological catalysts for electrochemical N2 reduction toward NH3 under ambient conditions. ACS Catal. 2019, 9, 5245 10.1021/acscatal.9b00994.

[ref123] Sahoo S. K.; Heske J.; Antonietti M.; Qin Q.; Oschatz M.; Kühne T. D. Electrochemical N2 reduction to ammonia using single Au/Fe atoms supported on nitrogen-doped porous carbon. ACS Appl. Energy Mater. 2020, 3, 10061 10.1021/acsaem.0c01740. 33134880 PMC7592340

[ref124] Abghoui Y.; Garden A. L.; Howalt J. G.; Vegge T.; Skúlason E. Electroreduction of N2 to ammonia at ambient conditions on mononitrides of Zr, Nb, Cr, and V: A DFT guide for experiments. ACS Catal. 2016, 6, 635 10.1021/acscatal.5b01918.

[ref125] Yao C.; Guo N.; Xi S.; Xu C.-Q.; Liu W.; Zhao X.; Li J.; Fang H.; Su J.; Chen Z.; et al. Atomically-precise dopant-controlled single cluster catalysis for electrochemical nitrogen reduction. Nat. Commun. 2020, 11, 4389 10.1038/s41467-020-18080-w. 32873783 PMC7463028

[ref126] Musie G.; Farmer P. J.; Tuntulani T.; Reibenspies J. H.; Darensbourg M. Y. Influence of Sulfur Metalation on the Accessibility of the NiII/I Couple in [N, N ‘-Bis (2-mercaptoethyl)-1, 5-diazacyclooctanato] nickel (II): Insight into the Redox Properties of [NiFe]-Hydrogenase. Inorg. Chem. 1996, 35, 2176 10.1021/ic9515968. 11666411

